# Harnessing Machine Learning Approaches for the Identification, Characterization, and Optimization of Novel Antimicrobial Peptides

**DOI:** 10.3390/antibiotics14121263

**Published:** 2025-12-14

**Authors:** Naveed Saleem, Naresh Kumar, Emad El-Omar, Mark Willcox, Xiao-Tao Jiang

**Affiliations:** 1 Microbiome Research Centre, St. George and Sutherland Clinical Campuses, School of Clinical Medicine, Faculty of Medicine & Health, University of New South Wales (UNSW), Sydney, NSW 2052, Australia; n.saleem@unsw.edu.au (N.S.); e.el-omar@unsw.edu.au (E.E.-O.); 2 School of Chemistry, University of New South Wales (UNSW), Sydney, NSW 2052, Australia; n.kumar@unsw.edu.au; 3 School of Optometry and Vision Science, University of New South Wales (UNSW), Sydney, NSW 2052, Australia

**Keywords:** artificial intelligence (AI), antimicrobial peptides (AMPs), databases, deep learning (DL), language models (LMs), multi-drug resistance (MDR), machine learning (ML)

## Abstract

Antimicrobial resistance (AMR) has become a major health crisis worldwide, and it is expected to surpass cancer as one of the leading causes of death by 2050. Conventional antibiotics are struggling to keep pace with the rapidly evolving resistance trends, underscoring the urgent need for novel antimicrobial therapeutic strategies. Antimicrobial peptides (AMPs) function through diverse, often membrane-disrupting mechanisms that can address the latest challenges to resistance. However, the identification, prediction, and optimization of novel AMPs can be impeded by several issues, including extensive sequence spaces, context-dependent activity, and the higher costs associated with wet laboratory screenings. Recent developments in artificial intelligence (AI) have enabled large-scale mining of genomes, metagenomes, and quantitative species-resolved activity prediction, i.e., MIC, and de novo AMPs designed with integrated stability and toxicity filters. The current review has synthesized and highlighted progress across different discriminative models, such as classical machine learning and deep learning models and transformer embeddings, alongside graphs and geometric encoders, structure-guided and multi-modal hybrid learning approaches, closed-loop generative methods, and large language models (LLMs) predicted frameworks. This review compares models’ benchmark performances, highlighting AI-predicted novel hybrid approaches for designing AMPs, validated by in vitro and in vivo methods against clinical and resistant pathogens to increase overall experimental hit rates. Based on observations, multimodal paradigm strategies are proposed, focusing on identification, prediction, and characterization, followed by design frameworks, linking active-learning lab cycles, mechanistic interpretability, curated data resources, and uncertainty estimation. Therefore, for reproducible benchmarks and interoperable data, collaborative computational and wet lab experimental validations must be required to accelerate AI-driven novel AMP discovery to combat multidrug-resistant Gram-negative pathogens.

## 1. Introduction

The increasing prevalence of antibiotic resistance worldwide has underscored the urgent need for the development of alternative novel therapeutic approaches [1,2]. There are fewer peptide-based antibiotics, such as polymyxins and bacitracin, and the majority of naturally occurring and engineered AMPs have not progressed to market because of poor pharmacokinetics and pharmacodynamics (PK/PD), and delivery limitations [3,4]. Antimicrobial peptides (AMPs) are one class of novel antimicrobials, but are yet to be fully commercialized as antimicrobials, as only a limited number have reached from bench to bedside [3,4].

Computational intelligence approaches, notably through machine and deep learning methods, have emerged as essential resources for novel AMP identification, characterization, and optimization, driving different predictive models, thereby enhancing the discovery processes for novel peptides [5,6,7,8]. Currently, these are being utilized not only to predict novel AMPs but also to address PK/PD concerns [9].

Machine learning algorithms have existed for several decades [10]. However, their nature and complexity have evolved significantly from conventional statistical approaches to deep learning architectural frameworks [10,11]. Recent advancements in computational resources and the development of powerful graphics processing units (GPUs) can efficiently handle the processing needs of complex machine learning approaches [10,11]. Moreover, the digital age has led to the availability of larger datasets worldwide, thereby providing useful resources to train data-hungry algorithms effectively [12,13].

**Figure 1:**
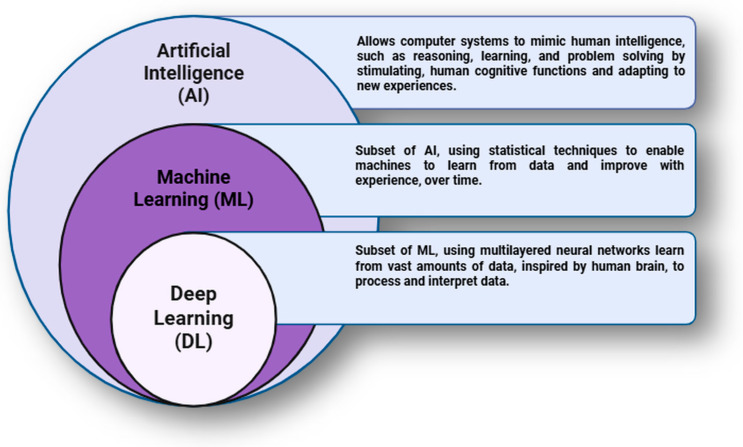
Relationships between artificial intelligence (AI), machine learning (ML), and deep learning (DL). AI facilitates the broad goal of building intelligent systems. At the same time, ML (AI subset) learns patterns from data, and DL (ML subset) powers modern ML pipelines within the larger AI toolkit for mining, identification, characterization, and optimization of novel AMPs. The illustration was created with BioRender (https://www.biorender.com, accessed on 12 November 2025).

Integration of artificial intelligence (AI) for antimicrobial peptide (AMP) discovery, along with conventional drug development methods, has shown a significant evolution within the field of computational biology and biomedicine (Figure 1) [5,6,7,8]. Various sophisticated machine learning models are also available [14]. By using these computational approaches, researchers have uncovered effective and rapid strategies for the prediction, identification, characterization, and optimization of novel AMPs exhibiting strong antimicrobial properties to combat drug-resistant pathogens [6,14].

AI models can explicitly predict ADMET properties (i.e., absorption, distribution, metabolism, excretion, and toxicity profiles) to guide sequential optimization and drug-dosing strategies [9,15]. Certain deep learning and transfer-learning models can accurately predict the in vivo half-lives of AMPs and highlight rational chemical and structural modifications to minimize systemic adverse events [9,15]. Reinforcement-learning approaches can enforce multi-objective features, so the proposed novel peptides can simultaneously satisfy desired bioactivity and conventional antibiotics’ PK/PD characteristics [16].

Currently available extensive database resources, combined with innovative computational methodologies integrating advanced predictive models, can pave the way for AMP exploration [17,18,19]. Machine learning models have evolved recently and fundamentally reshaped the landscape for drug discovery, allowing researchers to conceptualize drug design (Figure 1) [17,18,20,21]. Moreover, deep learning algorithms can analyze vast peptide sequence spaces, thereby predicting and optimizing peptide sequences to have better efficacy and safety (Figure 1) [21].

The horizon of AMP research can be broadened by multidisciplinary collaborations, leveraging computational biology, bioinformatics, medicinal chemistry, microbiology and drug development [22,23]. This allows for the potential for fully automated peptide-based drug discovery processes through AI to be used to develop highly selective and targeted antimicrobial therapies that could mitigate different infectious diseases [21,22,23]. As AI continues to revolutionize basic understanding and ability to manipulate peptide sequences, the field is standing on the precipice of achieving unprecedented advances in medicine to combat global life-threatening challenges associated with antimicrobial resistance [23,24].

AMPs can shift from “discovered” molecules to programmed novel therapeutics using different foundation approaches and closed-loop wet-lab validation [22,23]. This review highlights the latest advancements and opportunities associated with AMP mining, using various discriminative and generative AI-based models, and outlines the potential challenges and future directions. Moreover, it illustrates various currently available data resources, descriptors, and state-of-the-art conventional and deep machine learning models for identifying novel candidates and for assessing their stability, bactericidal, and toxicity assays, which are essential for AI-driven AMP designs.

Foundation models, such as transformers, diffusion, and large language models, can learn richly structured sequence-based functions rather than hand-coded patterns, shifting single-objective classifiers more toward controllable generators, to propose novel candidates under explicit constraints [21,22,23]. Moreover, foundation models highlight multi-model objectives, i.e., optimizing minimum inhibitory concentration (MIC) while improving protease stability and minimizing hemolysis and aggregation resistance, essential features that must be considered for translational readiness.

Therefore, this review proposes a conceptual and structural taxonomy of different ML approaches with minimal timeline context for AMP discovery, tailored for computational biologists, microbiologists, and physicians, showing interdisciplinary translation.

## 2. Conceptual Taxonomy of AI Methods for AMP Discovery

To capture the breadth of the rapidly expanding research on different machine learning approaches for antimicrobial peptide (AMP) discovery, a structured literature search across major primary scientific databases (PubMed, Google Scholar, EMBASE, Scopus, and Web of Science) was performed. The search encompassed papers published between January 2005 and September 2025 to reflect recent methodological advances in available machine learning, deep learning, and artificial intelligence to predict and optimize AMPs. Keywords and Boolean operators were used: “antimicrobial peptides” AND (“machine learning” OR “deep learning” OR “artificial intelligence” OR “neural networks” OR “transformer” OR “graph neural network” OR “language model”). Additional specific terms such as “evolutionary algorithms,” “AMP design,” “MIC prediction,” and “toxicity prediction” were used to identify relevant studies.

Abstracts and full texts were screened to prioritize peer-reviewed studies reporting different novel ML and DL architectures, and comparative benchmarking, alongside experimental validations, e.g., in vitro minimum inhibitory concentrations (MICs) assays, stability and cytotoxicity studies, and in vivo infection models. In total, several hundred studies were evaluated. Therefore, representative studies demonstrating translational relevance have been included to provide an overview of current methodologies and to reflect the diversity of computational strategies, ranging from classical discriminative models to innovative multi-modal and generative frameworks, with emphasis on translational relevance by highlighting their experimentally validated findings where applicable.

## 3. Designing AMPs

**Figure 2:**
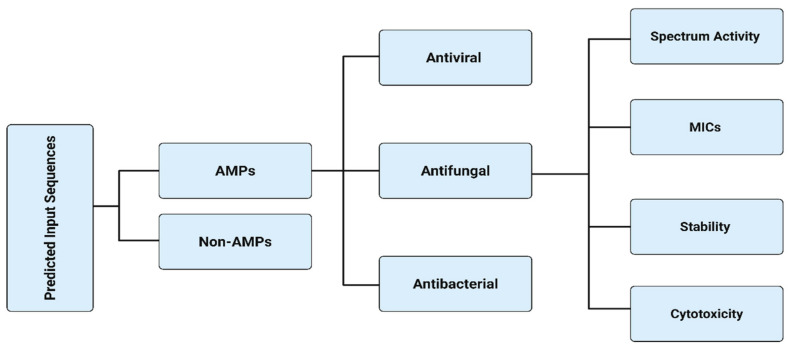
A hierarchical diagram for AMP discovery. Firstly, the input sequences must be differentiated between AMPs and non-AMPs to predict the spectrum of activity (viruses, fungi, and bacteria). Once novel AMP sequences are predicted, they can be accompanied by more detailed information on their activity spectrum, minimum inhibitory concentrations (MICs), stability, and potential cytotoxicity.

Various computational approaches can be used to discover novel AMPs using inputs containing short amino acid sequences and to determine antimicrobial activity by predicting the minimum inhibitory concentrations (MICs) required to inhibit or kill the growth of various microbes [25,26] (Figure 2). The amino acid input sequences can be classified as antibacterial, anti-viral, or anti-fungal, depending on their bioactivity [25,26,27]. The majority of validated AMPs range from 5 to more than 100 amino acids, with optimal physicochemical characteristics rarely preserved beyond this sequence limit [27,28].

**Figure 3:**
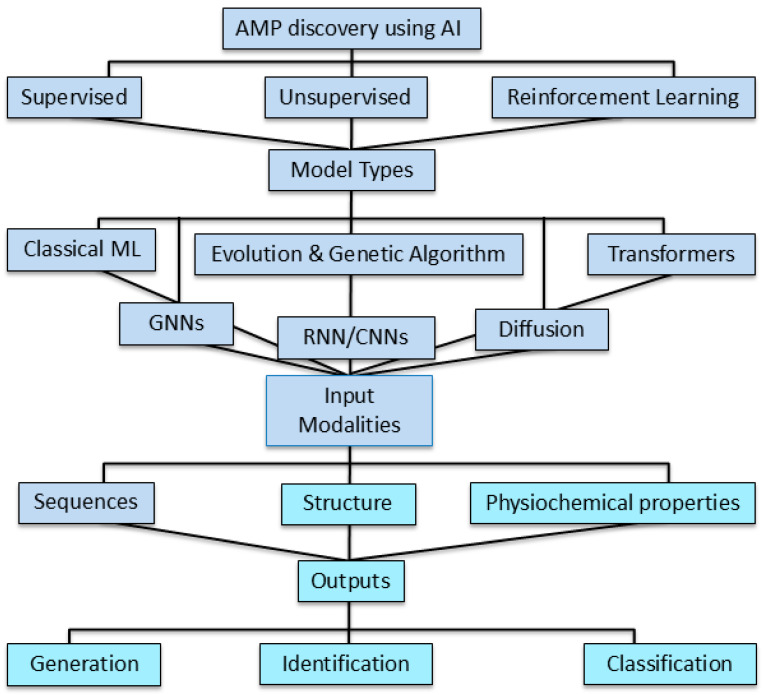
Different machine learning approaches for AMP discovery. The schematic layered learning paradigms comprise different machine learning methods (supervised, unsupervised, and reinforcement learning), model types (classical and deep machine learning approaches), input modalities (sequence, structural, and physicochemical properties), and tasks (classification and generation).

Machine learning classifiers are usually trained on AMP datasets having short-peptide windows, and long peptides are either generally poorly predicted or excluded due to functional and structural divergence (Figure 2 and Figure 3) [27,28]. Furthermore, the process allows higher taxonomical targeted classifications to be used to predict potentially microbe-specific minimum inhibitory concentrations (MICs), stability, and cytotoxicity [25,26,29,30] (Figure 2).

## 4. Learning Paradigms

AMP’s profiling can span multi-target structural and functional objectives, such as protease stability, potency, and non-hemolytic activity [9,28,31,32,33,34]. Learning paradigms can operate these targets, using supervised learning for physicochemical properties prediction, self-supervised language models for transferable embeddings, a reinforcement approach for goal-directed optimization, and active learning methods to close the loop alongside assays [9,28,31,32,33,34]. Altogether, this progression can turn proposed AMP designs into a data-driven and executable workflow.

Machine learning approaches are classified into three main categories [9,28,31,32,33,34] (Figure 3 and Table 1). Supervised learning approaches, such as classification and regression methods, utilize labeled data for classification and predicting AMPs’ microbial activity, either as binary outcomes or continuous minimum inhibitory concentrations (MICs) [9,28,31,32,33,34] (Table 1).

Unsupervised methods, such as representation learning and clustering of sequences, extract and predict novel AMPs with similar physicochemical profiles from unlabeled data by generating new sequences and learning peptide embeddings to identify hidden patterns, which supervised models may miss [9,28,31,32,33,34] (Table 1). The reinforcement learning approaches perform sequence design and optimization using reward loops through interactions within an environment and can iteratively refine peptides for maximizing the predicted activity [9,28,31,32,33,34] (Table 1).

In modern machine learning methods, AMP profiling has extended beyond mere non-AMP vs. AMP classifications with multi-targeted functional and structural objectives, such as species-specific membrane selectivity and activity, protease stability, safety risks, and immunomodulatory properties [35,36]. Integration of multi-objective profiling will allow ML approaches to prioritize candidates so that they demonstrate antibiotic-like potency with minimal toxicity and resistance potential, indicating how AI-guided AMPs will serve as novel alternatives therapeutic modalities to traditional antibiotics [35,36].

## 5. Task Categorization

Once learning paradigms are outlined to turn proposed AMP designs into executable models, task categorization can be performed to specify what these predicted models are trained to do [9,26,37]. This requires alignment with supervised classifiers to predict AMPs and their activity spectrum [9,37]. For characterization, systematic computational objectives map to regression and classification heads for stability, minimum inhibitory concentration (MIC), hemolysis, and optimization must be coupled with generative and evolutionary algorithms alongside active learning for goal-directed improvement [37,38]. Adopting framing tasks and multi-model approaches can make the predicted pipelines modular; each task can be plugged into the right paradigm, which favors multi-purpose AMP designs [37,38] (Table 2).

Classical machine learning approaches, such as Support Vector Machines (SVMs), Random Forest (RF), K-Nearest Neighbours (kNN), and decision tree tools, are used to identify and predict AMPs as binary outcomes based on pre-existing sequences and other attributes related to known AMPs [39,40] (Table 2). Deep learning (DL), i.e., Convolutional Neural Network (CNN) and Recurrent Neural Network (RNN) models, including Long Short-Term Memory (LSTM), predict sequence patterns, while transformer models adapt pretrained language models, e.g., ProteinBERT and PepNet, for the discovery of AMPs [39,40] (Table 2).

Graph Neural Networks (GNNs) are based on sequence embeddings and structural residue contact graphs for integrating both modalities for AMPs’ prediction and activity spectrum, whereas generative and diffusion models, such as generative adversarial networks (GANs), variational autoencoders (VAEs), and diffusion frameworks, are based on de novo AMP design, generation, and sequence optimization [39,40,41]. This classifies identification into discriminative/predictive and generative approaches, illustrating that supervised machine learning (ML) and deep learning (DL) can be used for AMP identification [39,40,41].

Generative models, such as generative adversarial networks (GANs), variational autoencoders (VAEs), diffusion models, genetic algorithms, and large language models (LLMs), can tackle the generation and refinement of guided AMPs [39,40,41]. Generative models such as generative adversarial networks (GANs), and variational autoencoders (VAEs) can learn through data-driven processes before sampling novel sequences, such as AMPGAN and HydrAMP [42,43]. Therefore, these models support latent space edits toward desired physicochemical and functional features [42,43] (Table 2).

Diffusion models, i.e., RFpeptides for macrocycles and Chroma, allow for programmable conditioning, thereby predicting target-aware AMPs’ designs [44,45], while genetic algorithms can facilitate goal-directed optimization under multi-targeted properties, e.g., better efficacy and safety profiles, and large language models (LLMs) like ESM3 can provide promptable sequences alongside structural and functional reasonings for guiding and refining novel AMP prediction [46,47] (Table 2).

## 6. Data Modalities

Inputs can be viewed based on a multimodal taxonomy [37,48,49]. 0D are zero-dimensional, physicochemical scalars alongside assay metadata, i.e., Data Repository of Antimicrobial Peptides (DRAMP) and Database of Antimicrobial/Cytotoxic Activity and Structure of Peptides (DBAASP), which supervise identification and characterization baseline features [49,50,51]. Therefore, 0D modality can be used for initial screening based on global descriptors for fixed-length vectors of whole peptides, including isoelectric points, overall net charge, and fraction of hydrophobic residues [49,50,51] (Table 3). However, first-generation style modality can lose local motifs and residual orders, resulting in it being worse at capturing contexts and long-range interactions [49,50,51].

The 1D modality is one-dimensional; this second-generation classic machine learning approach uses linear amino acid sequences, per-residue features, and sequence encodings such as one-hot, k-mer embeddings (ProtBERT, ESM), and language-model embeddings for robust classifiers and regressors to capture local motifs and periodicity by combining with classical engineered features, thereby limiting modeling of nonlocal 3D contacts (Table 3) [49,50,51].

The 2D modality illustrates pairwise two-dimensional approaches, adding evolutionary information within machine learning models, bridging engineered features and deep models as an intermediate, which can approximate inputs to Convolutional Neural Network (CNN) and Graph Neural Network (GNN) models for structural constraints by capturing topology for activity and toxicity without full 3D outputs (Table 3) [49,50,51].

The 3D modality runs and computes 3D structure-based descriptors such as amphipathic moments and surface hydrophobicity using structural and informed Graph Neural Networks (GNN) that operate on coordinates to directly model geometry and physics for novel candidates, thereby predicting 3D structures such as AlphaFold and Rosetta. The 3D modality supports structurally aware characterization and optimization, while generators, such as LLMs and diffusion, are conditioned either on 1D, 2D, or 3D cues and perform in silico screening before assays (Table 3) [49,50,51,52].

Therefore, multi-modal integrative models combining sequence and predicted structural approaches must be used for richer training and predictive modeling, thereby mapping modalities onto identification, characterization, and goal-directed design [52].

## 7. Database Sources and Representation

There is a need for comprehensive databases and data heterogeneity, such as different assays, and conditions must be standardized with careful curation for machine learning (ML). This has been fulfilled by several initiatives such as UniProt, SwissProt (generic proteins), and other AMP-specific databases such as Antimicrobial Peptide Database-3 (APD3), Collection of Anti-Microbial Peptides (CAMP), Database of Antimicrobial/Cytotoxic Activity and Structure of Peptides (DBAASP), Hemolytik, Linking Antimicrobial Peptides (LAMP), and structural data in the PDB [49,53] (Table 4). These repositories have not only provided essential information regarding the structure and physicochemical properties of peptides but also facilitated structure–activity relationships, highlighting their efficacy and safety profiles [49,53]. Moreover, this in-depth resource has allowed researchers to model and design novel peptides with tailored characteristics, thereby improving the efficiency of discovering therapeutic candidates with fewer cytotoxic events [49,53].

Databases such as Collection of Anti-Microbial Peptides (CAMP) have utilized several machine learning tools for predicting peptide activity, with accuracy rates up to 93%, illustrating the potential of AI-driven algorithms for guided peptide synthesis and experimental validation [54,55]. For translational impacts, CAMP must be paired with richer, assay-resolved sources, such as the Data Repository of Antimicrobial Peptides (DRAMP), and Database of Antimicrobial/Cytotoxic Activity and Structure of Peptides (DBAASP), alongside layered language-model embeddings or structure-aware filters, facilitating CAMP entries to become controllable seeds for multi-targeted designs [54,55].

Other repositories and databases, such as the Antimicrobial Peptide Database (APD), Data Repository of Antimicrobial Peptides (DRAMP), and Database of Antimicrobial/Cytotoxic Activity and Structure of Peptides (DBAASP), have become a crucial component of the computational landscape, providing comprehensive data resources which allow researchers to derive meaningful insights concerning structure–activity relationships (SARs) of different AMPs [56,57]. Moreover, these repositories facilitate the identification and prediction of novel candidates with strong bactericidal activity depending on common physicochemical properties and bioactivity trends.

Although some AMP classifiers have reported accuracies higher than 90%, these may be artificially overrepresented due to the availability of either homologous or duplicate peptide sequences present within major AMP repositories [13,56,58,59]. Sequential redundancy allows models to memorize patterns, which may affect benchmarking and performance metrics [60,61,62]. Therefore, not removing homologous sequences between testing and training datasets can downgrade true predictive metrics by up to 50–60%, especially for highly conserved AMPs [61,63,64].

To obtain reliable and meaningful estimates of performance metrics, the introduction of homology reduction tools and sequence deduplication is advisable before model training [60,61]. Algorithms such as MMseqs2 can provide highly sensitive clustering, which is suitable for AMP-centric databases, and CD-HIT can be used to cluster larger peptide datasets at defined identity thresholds; therefore, it can be used to remove redundancy, thereby reducing interpretational bias and preventing data leakage [60,61,62,63,64]. Incorporating these tools into the AMP pipeline strategy can ensure accurate metrics, thereby reflecting true generalization [60,61,62].

For better therapeutic effect and safety of peptides and to enhance their bioavailability and stability, the Database of Antimicrobial/Cytotoxic Activity and Structure of Peptides (DBAASP) provides detailed molecular dynamics (MD) models for over 3200 AMPs, demonstrating the synergy between computationally predicted models and experimental validations by performing MD analysis [49]. These models can facilitate structure-based drug designs, thereby allowing the rational modification of peptides to optimize specific interactions with microbial outer membranes [49].

Training on curated positive or negative datasets, which are either incorrectly labeled or biased against negative peptides, can inflate performance [65]. Careful considerations must be given to initiatives such as AMPBenchmark for appropriate dataset splitting [66]. Early classical models used hand-crafted features, including amino acid composition, overall charge, and hydrophobicity [5,6,7,8]. Current modern deep learning methods learn about these characteristics directly via embeddings, such as sequence representations, including NLP embeddings from protein LMs (ProtBERT, ESM-2), one-hot vectors, and autoencoder features [39,40,41,65].

Structural representation usually uses graphs such as nodes, residues, and 3D convolutions on voxel grids [37,41,48]. Therefore, incorporating different features within frameworks such as SSFGM (Sequence, Structure, Surface, Graph, and Geometric-Based Model), including combined sequences, structural, and surface properties, could significantly outperform sequence-based baselines, thereby advancing the sequence, structural, and functional paradigms for accurate AMP prediction [37].

**Table 4:** Currently available AMP databases.

Database	Key Features
**a. Sequential search and AMP discovery databases**	
ADAM 2015 (A Database of Anti-Microbial Peptides)	Focused on AMPs’ structural classification with distinctive structural fold clusters, linking sequences to structural folds [67,68,69].
AMPDB V1 2023 (Anti-Microbial Peptide Database Version 1)	Integrated tools, i.e., MSA, BLAST, and AMP physicochemical feature calculators are incorporated within the databases [70].
AMPsphere	Possessed pan-kingdom collections, alongside advanced search filters depending on AMP properties [71,72,73,74].
APD and APD-3 2004 (first version) (Antimicrobial Peptide Database)	Possessed natural AMP features such as taxonomy, modification, and activity. APD-3 has annotated 3D structures [75].
BactiBase	Emphasized on ribosome-synthesized peptides, molecular docking inputs, 3D structures, and bacteriocin-class-specific models [76].
B-AMP (Biofilm-AMP) 2021	3D protein-peptide interactive models and preference scales predicted AMPs for in vitro, in vivo, and in silico methods [77].
CAMP R3, R4, and R5 2010 (first version) (Collection of Anti-Microbial Peptides)	Predicted secondary structures and added metagenome-derived AMPs from human gut microbiomes [78,79].
DBAASP v3 and v4.1 2010/2020 (Database of Antimicrobial/Cytotoxic Activity and Structure of Peptides)	Predicted 3D-based models alongside bactericidal activity and cytotoxicity [49].
DRAMP 3.0 and 4.0 2022 (first version) (Data Repository of Antimicrobial Peptides)	Based on synthetic derivatives, the latest version focused on clinical translation, adding stability and cytotoxicity data [50,56,80].
dbAMP and dbAMP 2.0 2019 (first version) (Database of Antimicrobial Peptides)	Incorporated proteomics and transcriptomics-derived AMPs having post-translational modification sites [81].
InverPep (Invertebrate Peptides Database)	Possessed curated host defense peptides with bactericidal activity against MDR pathogens from invertebrates [82].
LAMP2 (Linking Antimicrobial Peptides-2)	Used metagenomes and BLAST (https://blast.ncbi.nlm.nih.gov/Blast.cgi, accessed on 12 November 2025), alongside a Python API [83].
MilkAMP	Linked to an external database (UniProt) and predicted dairy-related AMPs and bactericidal activities [84].
MLAMP	Provided standardized splits for cross-validation [85].
modlAMP 2020 (Molecular design laboratory’s Antimicrobial peptides)	Python-based software offered molecular descriptors and amino acid sequences by providing access to completed datasets [86,87].
Peptipedia v2.0	Comprised peptide databases, alongside built-in predictive models based on their activity [88].
Pep Bank 2012 dbAMP, BIOPEP-UWM, YADAMP)	Aggregated commercial peptides and predicted MICs [89].
PhytAMP	Possessed plant-derived AMPs, specialized for phytoalexins and defensins [90].
PlantPepDB	PhytAMP integrated physicochemical properties to predict tertiary structures for therapeutics discovery [91].
YADAMP 2012 (Yet Another Database of Antimicrobial Peptides)	This dataset is searchable by amino acid name, number, net charge, and sequence motifs [92].
**b. Structural and physiochemical annotation databases**	
StAPD (Stability-Aware Peptide Database)	Predicted candidates for in vivo methods by integrating 3D structures such as AlphaFold and PDB [93].
DPL (Database of Peptide Ligands)	Possessed structural and targeted binding information essential to target AMP interactions [94].
modlAMP (structural module)	Provided computed physicochemical profiles [86,87].
**c. Stability, bioactivity, and cytotoxicity profiling databases**	
DBAASP v3 and v4.1 v3 (2018), v4.1 (2020)	Gold standard for structural MIC and cytotoxicity annotation [49].
B-AMP 2021	Biofilm inhibitory activity profiling [77].
mlAMP 2020 (toxicology module)	Safety profiling, i.e., hemolytic and cytotoxicity [86,87].
DRAMP 2020 (activity section)	Safety profiling, i.e., stability, MIC, toxicity, hemolysis [50,56,80].
BIOPEP-UWM 2020 (via Peptipedia/dbAMP)	Contains cytotoxic and enzymatic activity annotations [95,96].

## 8. Models for Novel AMP Mining and Discovery

### 8.1. Conventional Machine Learning Using Discriminative Models

#### Classical Models Based on Random Forests (RFs), Support Vector Machines (SVMs), and GBM Architecture

Conventional algorithms use AMP classifiers based on “handcrafted” features such as amino acid composition and physicochemical descriptors [39,97]. Current machine learning approaches, such as Random Forests (RFs), Support Vector Machines (SVMs), k-Nearest Neighbours (kNNs), and ensemble methods, can be used to predict novel AMPs [39,97].

These models can handle complex and high-dimensional biological data composed of hundreds of evolutionary, physicochemical, and sequence-derived descriptors [39,97]. Therefore, they capture meaningful sequences and functional relationships after training on diverse AMP datasets. By leveraging these rich feature spaces, RF, SVM, and GBM architectures can help to reduce overfitting and improve the robustness and accuracy of AMP classifiers [39,97] (Table 5). Accuracies have been reported for machine learning algorithms as high as 87.5% for Discriminant Analysis (DA), 91.5% with SVM, and 93.2% with Random Forests (RFs) on larger AMP datasets [98].

The latest tools, such as Integrated Antimicrobial Peptide Estimator (IAMPE) in combination with multiple algorithms, e.g., k-Nearest Neighbours (kNNs), Naïve Bayes, Support Vector Machines (SVMs), Random Forests (RFs), and XGBoost, based on composition and physicochemical features, achieve up to 95% accuracy by reducing individual model bias and capturing both structural and functional AMP patterns [39,99].

Conventional discriminative machine learning models have been widely applied to novel AMP mining and discovery. Models, such as RFs, SVMs, and Gradient-Boosting architectures, can classify sequential inputs derived from synthetic and commercial libraries, metagenomics, and proteomics, and identify novel peptides with higher probabilities of antimicrobial activity [100,101]. For instance, XGBoost-based iAMPCN and RF-based AMPEP frameworks were used to screen and optimize previously uncharacterized sequences, with top-ranked candidates for experimental validation against *E. coli* and *S. aureus* [100,101]. Therefore, classical models can play key important roles during the initial stages of AMP discovery pipelines by quickly analyzing larger datasets and prioritizing candidates for wet-lab validations [102,103,104].

Random Forests (RFs), Support Vector Machines (SVMs), Artificial Neural Networks (ANN), and Naive Bayes can effectively differentiate between non-antimicrobial and antimicrobial peptides using computational features derived from tertiary structures, as well as sequence-based features and physicochemical descriptors [105,106]. RF-based methods have proven to be superior for predicting peptide activity, with decision-tree-based algorithms demonstrating competitiveness for peptide classification tasks, acknowledging that different algorithms may excel under different circumstances [106].

Currently, several conventional discriminative models can facilitate the shortlisting of experimentally validated AMPs. Random-forest classifiers, i.e., AmPEP and related RF and SVM pipelines, were used to screen metagenomic open reading frames (ORFs), synthesize top-ranked candidates, and demonstrate inhibition of Gram-negative pathogens [107]. Moreover, these models operated by using sequence-derived physicochemical descriptors, making them ideal for in vitro wet-lab validation, adopting FASTA files and commercially available AMP web servers and databases [107]. Microbiologists simply upload peptide FASTA files to pre-trained AMP predictors, such as AmPEP, AmpGram, and CAMP-R3. Therefore, these servers can automatically run classification and feature extraction in the background, predicting top-ranked AMP candidates, which must be synthesized and evaluated experimentally without local scripting and model retraining [107].

Gradient-boosting decision models, such as XGBoost and LightGBM, as strong baselines for tabular, heterogeneous AMP features, build an additive ensemble of shallow trees that iteratively fit residuals, with regularization, fast split finding, and either ordered or categorical handling [108]. An ensemble framework combining LightGBM classifiers alongside Convolutional Neural Networks (CNNs) integrated sequential, structural, and physicochemical features, demonstrating superior predicted performances over pre-existing conventional methods [109]. AMPpred-EL combined with LightGBM and logistic regression distinguished AMPs from non-AMPs, and outperformed state-of-the-art approaches on benchmark datasets, showing better accuracy and efficiency outputs [110].

Well-tuned classical models predict with up to 90% accuracy upon cross-validation, while comparing known and unknown AMPs [23,39]. These models use either reduced alphabets or amino acid counts as inputs, and their performances depend on training data types and negative controls [23,39]. Traditional machine learning models can also be evaluated in regression mode [23,39]. Moreover, the latest studies showed that gradient-boosting regressors and RF based on quantitative structure–activity relationship (QSAR) features achieved Pearson correlations of 0.70 to 0.74 and mean squared errors (MSEs) of 0.34 to 0.39 for predicting log-MIC against *E. coli* [111]. Therefore, classical ML can provide estimations regarding potency (regression) alongside binary classification [23,39].

**Table 5:** Different currently available machine learning models using discriminative methods.

Models	Architectural Framework	Performance Metrics	Key Features and Predicted Properties
**a. Classical ML (RF, SVM, GBMs)**			
AntiBP2 and AntiBP3	SVM	AntiBP2: Accuracy (92.1%), MCC (0.84), AntiBP3: AUC (0.93–0.98), (MCC up to 0.86)	SVM-based predictors used balanced +ve and −ve training datasets using residue contact maps integrated with in silico toxicity screening. Predicted antimicrobial activity [85,112].
AmpClass	Ensemble ML (XGBoost, RF, NN, DT)	Accuracy (93.2%)	Classification and regression approaches to predict novel AMPs. Predicted antimicrobial activity [108].
AmpGram	N-gram encoding and stacked random forests.	AUROC (0.98)	Predicted longer peptides (>10 A.A), used for high-throughput proteomics-based AMPs. Predicted antimicrobial activity [113].
AmPEP	RF classifiers	Accuracy (96%), MCC (0.90), AUC (0.99)	Provided distribution patterns of amino acid features, with higher accuracy, simplicity, and reduction capability. Predicted antimicrobial activity [114].
AmPEPpy	Random Forest (RF)	Accuracy (91%)	It predicted plant peptides using amino acid composition features [115,116].
CalcAMP	LightGBM, ensembleand tree algorithms	Accuracy 86–90% (best RF model 90%)	It used a curated dataset of validated short AMPs and classified them based on their spectrum. Predicted antimicrobial activity [117].
CAMP-R3/R4	ML classifiers (SVM, RF, DA, HMM)	R3: Accuracy (90.5%), R4: AUROC (0.93)	Multi-model approaches showed improvement in performance, i.e., area under the receiver operating characteristic curve. Predicted antimicrobial activity [78,79,85,118,119,120,121,122].
iAMPpred (AMPredict)	SVM	Accuracy (74–88% depending on class)	It used physicochemical descriptors like hydrophobicity and charge. Predicted antimicrobial activity [123,124].
iAMP-2L	Two-layered FKNN with PseAAC	Overall accuracy (87.6%)	Multi-model approach predicted five function categories. Predicted antimicrobial activity [125].
MLAMP (Multi-Label AMP predictor)	Ensemble of different ML, i.e., SMOTE and PseAAC	Micro-F1 (0.78)	Multi-label peptide functional models identified AMPs and their biological roles [126].
MLBP	Multi-scale ML alongside CNN, and BiGRU	Accuracy (86%)	It processed raw sequential vectors without having LLM inputs. Predicted antimicrobial activity [127,128].
PEPred-Suite	Different RF models	Accuracy (89%), AUC (0.92)	Using sequence-based descriptors and adaptive learning to predict efficacy and safety profiles [129].
Target-AMP	RF, SVM, KNNs	Accuracy (93.8%)	Used evolutionary data and composition features with multiple classifiers to predict AMP. Predicted antimicrobial activity [130].
**b. Deep learning (CNN, GNN, RNN, transformers, others)**			
AI4AMP	CNN, LSTM, DNNs	Accuracy (91.7%)	Used PC6 and autocovariance to predict novel AMPs. Predicted antimicrobial activity [131].
AMPlify	Bi-LSTM, Multi-head attention using Word2Vec tokens	AUROC (0.984), AUPRC (0.986). F1 (0.94)	An attention-based model to validate against the WHO priority pathogens using ensemble learning to improve robustness [132,133].
AMPpred-CNN	1D CNNs	Accuracy (92%)	Peptide sequences were encoded using CTD descriptors to predict AMPs. Predicted antimicrobial activity [134].
AMP Scanner AMP Scanner v2	CNN, RNN, deep neural network	Accuracy (92%), MCC (0.85)	Predicted efficacy based on physicochemical properties. Predicted antimicrobial activity [39,135].
APIN	CNN with embedded layers	Accuracy (94%)	Predicted AMPs using convolutional architectures directly from sequences. Predicted antimicrobial activity [136].
deepAMPNet	Pretrained Bi-LSTM, GNNs, and structural graphs	AUROC (0.97)	Alongside structure and language-derived encodings, it predicted delivering accuracy and biological insights. Predicted antimicrobial activity [137].
Deep-AmPEP30	CNN trained on PseKRAAC for shorter peptides	Accuracy (92.6%)	Optimized and predicted shorter peptides [138,139,140].
DMAMP	CNN, Residual CNN Blocks, PSSM	Accuracy (91.3%)	Multi-task predictive model used CNN–residual architecture and evolutionary features fusion for robust and accurate prediction. Predicted antimicrobial activity [141].
HDM-AMP	ESM-1b, Deep Forest	Accuracy (89.5%)	It was interpreted and predicted using an ensemble of decision trees [142].
iAMPCN	CNN	Accuracy (93.4%)	It used handcrafted features and classified using CNN without LLM embeddings. Predicted antimicrobial activity [143,144].
iAMP-CA2L	CNN, Bi-LSTM, and SVM	Accuracy (91.2%)	It used hybrid SVM models for final classification with a dual-task focus [145,146].
LMPred	ProtTrans, CNN classifiers, T5, and XLNet	Accuracy (92–93%)	Bridged protein sequence understanding and learned pattern detection. Predicted antimicrobial activity [147].
MBC-attention	CNN, Attention, ML	MCC (0.81)	It focused on critical residual motifs causing membrane disruption using attention models. Predicted antimicrobial activity [148,149].
sAMPpred-GAT	Graph Neural Network (GAT) and ML	AUROC (0.95)	Graph attention networks leverage sequence-to-graph conversion [150].
TP-LMMSG	Deep learning GNN on proteins, LM residues	Accuracy (94%)	Protein-LM, based on each residue, provided multi-scaled structural information. Predicted antimicrobial activity [151].
**c. Ensemble models with hybrid frameworks**			
AMP-BRET	RF, ProtBERT Transformer with fine-tuning for regression	Accuracy (92.1%)	Demonstrated high precision and transfer learning from protein corpora. Predicted antimicrobial activity [152,153].
AMPpred-EL	Ensemble ML (Logistic Regression and LightGBM)	Accuracy (93.8%)	It combined multiple ML components for stronger AMPs prediction. Predicted antimicrobial activity [110].
AMPpredMFA	LSTM, CNN, Attention, and MLP	AUROC (0.97)	It integrated local (CNN) and long-range (LSTM) sequence features to predict features [154].
AMP-META	Light GBM (LGBM) comprises different AMP tools	Accuracy (95%)	It predicted physicochemical descriptors using larger datasets. Predicted antimicrobial activity [155,156].
E-CLEAP	Ensemble of four MLP classifiers	Accuracy (97.3%) (AAC features), Accuracy 84.0% (PseAAC features), F1 (0.93)	Ensemble neural classifiers performed high-accuracy AMP classification. Predicted antimicrobial activity [157].
**d. Transformer-based models**			
AMPTrans	LSTM, transformer, RF, SVM with adaptive QSAR	Accuracy (93%)	QSAR-enabled designer facilitated sequences guidance and novelty [158].
AMP-ProtBERT	Fine-tuned ProtBERT	AUROC (0.98)5 (ProtBERT AMP classifier), Accuracy (94%)	iAMP-bert (ESM-2) pretrained models outperformed for AMPs prediction and antimicrobial activity [153,159,160].
**e. Specialized supportive models**			
ESKAPEE-MICpred	LSTM, CNN, and MLP	R2 = 0.82 (Species-specific MIC regression model)	It used sequence-derived descriptors to predict activity [161].
EnDL-HemoLyt	LSTM, CNN, and MLP	AUROC (0.97), MCC (0.80)	It optimized the therapeutic index by predicting hemolysis [24,162].
panCleave	RF, predicted protease cleavage sites	AUPRC (0.92)	panCleave to predict in vitro and in vivo efficacy and safety using the proteome of extinct species [163].
StaBle-ABPpred	BiLSTM	AUROC (0.95); Accuracy (0.91), MCC (0.82), AUPRC (0.97)	It predicted peptides’ stability and activity using Word2Vec embeddings [164].
SMEP/SAMP	LSTM, XGBoost	Accuracy (90%), F1 (0.89)	Used different libraries such as nonapeptide, heptapeptide, and octapeptide to predict efficacy and safety [38,165,166].
**f. Data-centric ML**			
GMSC-mapper	Modified version of Prodigal, i.e., RF	Accuracy (89%)	It identified and annotated smaller proteins using microbial (meta)genomes [167,168,169].
Macrel	RF with metagenomics mining, LP models	AUROC (0.97–0.99 depending on datasets)	It predicted sequence-derived descriptors using proteome, genome, and transcriptome to show efficacy and safety [170,171].
**g. Emerging automated ML models**			
AutoPeptideML	AutoML-based peptide classifier with evolutionary features	Accuracy (90%)	It automated feature engineering from evolutionary indices to predict antimicrobial activity [172].
APEX	RNN, ATT, and MLP	Accuracy (92%)	Deep learning aided by molecular de-extinction to show in vitro and in vivo properties [26].
PrMFTP	Multiscale CNN, BiLSTM, multiheaded self-attention approach	Accuracy (93%), F1 (0.92)	It combined architectural models, i.e., CNN, BiLSTM, and self-attention, which were not derived from LLM [173].
**h. Other ML models**			
AMPActiPred	Multi-class ML functional	Accuracy (91%)	Three-stage framework employed peptide descriptors to capture compositional and physicochemical properties and activity [174].
Ansari & Colleagues	RNN, LSTM	AUROC (0.93)	Semi-supervised method predicted peptides efficacy and safety via positive-unlabeled learning [103,175].
Capecchi & Colleagues	RNN, GRU, SVM; MLP	AUC (0.95)	ML used DBAASP to predict short non-hemolytic and microbial activity of AMPs [176].
Zhuang & Colleagues	QSVM	Accuracy (94%)	It predicted sequence-derived descriptors to predict safety profiles [177].

### 8.2. Deep Learning Approaches Using Discriminative Models

Different AI-driven approaches, particularly machine learning (ML) and deep learning (DL), can be effective for predicting the antimicrobial potential of peptides, depending on their structural and physicochemical properties [105,106]. Through comparative performance evaluations, the efficacy of different machine learning and deep learning techniques can be confirmed [105,106]. Deep learning models can outperform better than conventional methods in some situations when compared to traditional feature-based approaches [103,175] (Table 5 and Table 6). These approaches suggest that confidence in these sophisticated computational methods has increased recently, especially when combined with transfer learning tactics that can improve forecast accuracy by using pre-trained models [176].

#### 8.2.1. Deep Learning with Recurrent Neural Networks and Recurrent Neural Network Frameworks

Machine learning methods can be employed for AMP prediction, involving the selection and integration of relevant features to enhance model accuracy. The integration of structural, compositional, and physicochemical characteristics into the Pseudo Amino Acid Composition (PSEAAC) improves predictive models for AMPs [178], thereby highlighting advanced deep learning modalities to enable precise AMP prediction and identification by leveraging comprehensive data resources [178].

Recurrent networks such as Recurrent Neural Networks (RNNs) and Long Short-Term Memory (LSTM) label AMPs as “sentences” of amino acid “words” [179]. LSTM-based classifiers can be pre-trained via Word2Vec and can capture sequence order [138,180]. For instance, Deep-ABPpred, using word2vec skip-gram, can be embedded into a bidirectional LSTM and distinguish AMPs correctly [138,180].

In general, Convolutional Neural Networks (CNNs) and Recurrent Neural Networks (RNNs) demonstrate similar performances for AMP detection, but Convolutional Neural Networks (CNNs) can be trained faster [41]. Convolutional Neural Network (CNN) models have been reported to have a Pearson correlation of 77% and an accuracy of 97% for MIC prediction [181]. Most deep classifiers are benchmarked on public AMP databases (e.g., APD3) and are better than Random Forests (RFs) and Support Vector Machines (SVMs), showing >90% accuracy [182,183].

To illustrate the practical aspects of deep discriminative models, the latest CNN and biLSTM architectures, such as AMP-BiLSTM and dsAMP, can be used to predict larger peptide designs [59,178]. In AMP-BiLSTM, deep sequential encodings trained on known AMP resources to predict top-scoring novel candidates, later validated experimentally against *E. coli* and *S. aureus*, achieved higher hit rates compared to conventional methods, demonstrating that different deep learning models must be directly incorporated within the design and test cycles [178].

Apart from standard classification methodologies, deep learning frameworks have been developed to enhance their predictive accuracy [184]. A model has been proposed that combines deep learning algorithms and different physicochemical properties to improve AMPs’ predictive capabilities [131]. Similarly, the AMPDeep hybrid model utilized transfer learning approaches, successfully predicting the AMPs’ hemolytic activity, thereby highlighting the potential of advanced learning algorithms for refining peptide screening [184]. All these innovative approaches may indicate a growing trend of integrating deep learning methods along with conventional ML approaches to improve the accuracy and robustness of AMP identification and prediction.

#### 8.2.2. Ensemble Models with Hybrid Frameworks

Hybrid and ensemble frameworks anchor AMP prediction by fusing complementary inductive biases across different models and features. AMP-BRET coupled RF alongside ProtBERT transformers fine-tuned for regression, achieving higher precision and pathogen-specific activity, estimated while leveraging transfer learning from large protein corpora [152,153]. The AMPpred-EL framework comprised logistic regression and LightGBM [110].

AMPpredMFA incorporated Convolutional Neural Networks (CNNs) due to local motifs, Long Short-Term Memory networks due to their long-range dependencies, and attention (context weighting) to capture diverse sequential and functional relationships underpinning multi-purpose activity [154]. AMP-META orchestrated LightGBM along with physicochemical descriptors and larger datasets, showing stronger generalization without hampering the interpretability of distinctive features [155,156].

The E-CLEAP ensemble of four Multilayer Perceptron Classifiers (MLP classifiers) demonstrated that tree-based methods, linear stacking, and neural learners improved robustness and calibration for AMP classification, particularly when heterogeneous assays and class imbalance could degrade single-model performance to distinguish between AMPs and non-AMPs, providing stable hit lists for screening [157]. AMPlify with attention-based biLSTM ensemble architectural frameworks scored metagenomic sequences, showing novel peptides exhibited lower MICs against WHO priority pathogens [133].

Amongst them, the practical considerations must be based on explicit uncertainty estimation, i.e., ensembles and bootstraps, and complementary error profiles, when trained with stratified and leakage-guarded splits [155,156]. Therefore, translational implications require the area under the curve (AUROC) and precision and recall (PR), alongside other parameters, such as species and strain-disjoint performance, calibration, MIC regression error, and hit enrichment, including ablation across ensemble components and failure analysis by sequence family [155,156].

#### 8.2.3. Transformer-Based Models

Deep learning models can apply transformer architectures from Natural Language Processing (NLP), such as AMP-READ models, AMP-CLIP, and Bidirectional Encoder Representations from Transformers (BERT) fine-tuned models [7,152,153,185]. BERT-AmPEP60, a ProtBERT fine-tuned transformer, predicted log-MIC against *Staph. aureus* and *E. coli*. BERT-AmPEP60 transcended classical regressors by achieving mean squared error (MSE) = 0.266 (vs RF’s 0.344) and a Pearson coefficient of r = 0.80 against *E. coli* [111]. The classification ML method, an ensemble of Convolutional Neural Network (CNN), Long Short-Term Memory (LSTM), and attention models, which was trained on gut microbiome peptides data, reported an accuracy of more than 83%, i.e., 11 novel AMPs demonstrated bactericidal activity against resistant Gram-negative bacteria and reduced lung infections >10-fold within an animal model [21].

For imbalanced datasets, UniAMP efficiently identified and predicted novel AMPs from microbiomes and leveraged inferred features using UniRep, ProtT5, and transformers by replacing manual features such as amino acid sequence composition and physicochemical properties, thereby outperforming baseline metrics such as the Matthews Correlation Coefficient (MCC) (0.9158) for pathogen-specific candidates, particularly against *P. aeruginosa* [58]. UniAMP mitigated manual features redundancy, thereby enhancing accuracy for large datasets and demonstrating potential overfitting for smaller datasets [58]. Therefore, it demands larger, diverse validation for proposed untested candidates to accelerate AMP pipelines but requires multi-mechanistic exploration to bridge in silico bench-side validations into clinical translation [58].

Transformer encoder frameworks, such as PeptideBERT and PepNet, exploited transformer-derived embeddings to predict peptides’ efficacy and safety by screening millions of peptides to produce many that had stronger bactericidal activity and lower cytotoxicity [186]. The latest tools, such as HMD-AMP and AmpHGT, embed peptides with ESM-2 to classify their antimicrobial properties, thereby highlighting pathogen-specific screening [142,187].

Transformer-based models have higher discriminative power when trained on large labeled sequential data by learning complex residue–residue dependencies [7,152,153,185]. In the presence of sufficient sequential data, these deep learning models can capture global contextual patterns, including evolutionary signatures, motif arrangements, and physicochemical gradients, thereby enhancing their ability to actively distinguish AMPs from non-AMPs [7,152,153,185].

The EvoGradient model used a deep learning in silico approach on oral microbiome peptides and generated 32 candidates, amongst which 6 AMPs showed enhanced bactericidal activity against *A. baumannii*, carbapenem-resistant *E. coli*, *K. pneumoniae*, and *vancomycin-resistant Enterococcus faecium* in vitro [188]. Novel candidates, i.e., pep-19-mod, were associated with reduced bacterial loads of 95%, targeting membranes without toxicity, following both topical and systemic administration within the animal model [188]. However, local optima risks and smaller datasets limited generalizability. Motif identification provided scaffold designs, but it requires contextual validation [188].

#### 8.2.4. Specialized Supportive Deep Learning Models

AMPs often have a broad spectrum of activity; therefore, understanding their physicochemical properties, such as overall structural configuration, hydrophobicity, and charge, is crucial [189]. Plisson and colleagues illustrated the significance of properties associated with the hemolytic activity, thereby reinforcing the notion that effective AMP designs must require an equilibrium of desired activity along with minimal cytotoxic events [189]. This highlights that there must be a delicate balance of efficacy and safety integral for peptide design, which AI can assist with by navigating through various rigorous predictive models.

By combining integrative approaches such as mutational scanning and machine learning, the mechanistic understanding of AMPs has progressed significantly [190]. Advanced screening techniques facilitated by AI have enabled the rapid exploration of peptides exhibiting improved selectivity, thereby reducing hemolytic activity [190]. All these advancements represent a significant leap from traditional screening methods, highlighting the efficacy of machine learning approaches for identifying promising candidates with desired physicochemical properties [190].

#### 8.2.5. Emerging Automated Machine Learning Models

Emerging automated models, i.e., AutoPeptideML, provide end-to-end auto machine learning pipelines and automatically facilitated model and feature selection, alongside homology-aware data partitioning [26]. They can predict novel peptides with adequate bioactivity using point-and-click workflows, thereby maintaining the library and emphasizing generalization beyond canonical residues and shipping production web and server stacks [172]. On the contrary, APEX operationalized “molecular de-extinction,” and trained deep sequence models, such as RNNs and attention layers with multitask heads, on extinct proteomes to identify and optimize novel peptides with prospective in vitro and in vivo validations, illustrating that automated discovery can be used to traverse unconventional sequence spaces [26].

PrMFTP targeted multi-functional therapeutic peptides using non-LLM deep architectures, i.e., Convolutional Neural Networks (CNNs), Bidirectional Long Short-Term Memory (BiLSTMs), and multi-head self-attention layered approaches, captured local motifs and long-range dependencies directly from raw sequences, with class-weighting to handle label imbalance datasets, and served as practical templates for automated machine learning back-ends to support multi-label endpoints [173,191]. Together, these models can automate curation and model selection, i.e., AutoPeptideML, and data mining with translational readouts, e.g., APEX, and multi-functional sequence learners (PrMFTP), and integration into closed-loop workflows for AMP designs [191].

#### 8.2.6. Regression Models

Beyond binary AMP identification, various deep learning models can predict quantitative antimicrobial activity [181]. A convolutional neural network concurrently classified AMP activity and predicted MIC values against *E. coli* using the Giant Repository of AMP Activities (GRAMPA) databases [164]. This network outperformed compared to different deep learning models and used open-source frameworks for computational AMP design and experimentally tested their efficacy against *E. coli*, *P. aeruginosa*, and *S. aureus* in vitro [128].

A significant gap within quantitative activity modeling has been filled by BERT-AmPEP60 based on refined Bidirectional Encoder Representations from Transformers (BERT) embeddings, and created transfer-learning regression models for MIC prediction against *S. aureus* and *E. coli* [111]. This model outperformed traditional machine learning and deep learning models, such as CNN-based MBC models and large protein-language models (ESM2), illustrating that Bidirectional Encoder Representations from Transformers (BERT) with transfer learning successfully predicted species-specific AMPs, employing conventional sequence characteristics [111,152,153]. Antimicrobial peptide recognition (ACEP) used classification and regression models, showing regression accuracy up to 97%, indicating that deep networks estimated potency after training on curated MIC databases [152,153,192].

In general, discriminative ML models demonstrate impressive performance, i.e., more than 90% accuracy for AMPs prediction using curated benchmarks. Deep architecture such as CNNs, RNNs, and transformers usually requires ample datasets and perform better than conventional models. However, a key limitation is generalization; most models are trained on similar data resources; therefore, their real-world hits can be lower upon cross-validation, and this must be minimized by experimentally testing predicted peptides.

To link regression approaches with microbiology, a MIC-focused model with an ensemble deep learning regressor predicted AMPs with lower MICs against key pathogens, thereby confirming stronger correlations between predicted and measured MICs [193]. Therefore, multi-purpose regression models can demonstrate both antimicrobial efficacy and hemolysis, enabling microbiologists to predict and optimize novel candidates with better efficacy and safety profiles before wet-lab testing [193].

Relevant metrics, i.e., MICs and activity spectrum, must be used for clinical relevance, rather than simpler classification accuracy. Studies such as BERT-AmPEP60 [111] and EvoGradient [7] used MIC-based metrics against ESKAPE pathogens [194]. Traditional ML models, such as RF and SVM, and modern deep learning approaches, i.e., CNNs, RNNs, and Transformer models, can achieve higher predictive accuracy using known datasets, and several have been validated by different in vitro and in vivo studies. Nevertheless, the current evidence has shown that AI-driven multi-model approaches can accelerate novel AMP discovery.

### 8.3. Large Language Models (LLMs)

**Figure 4:**
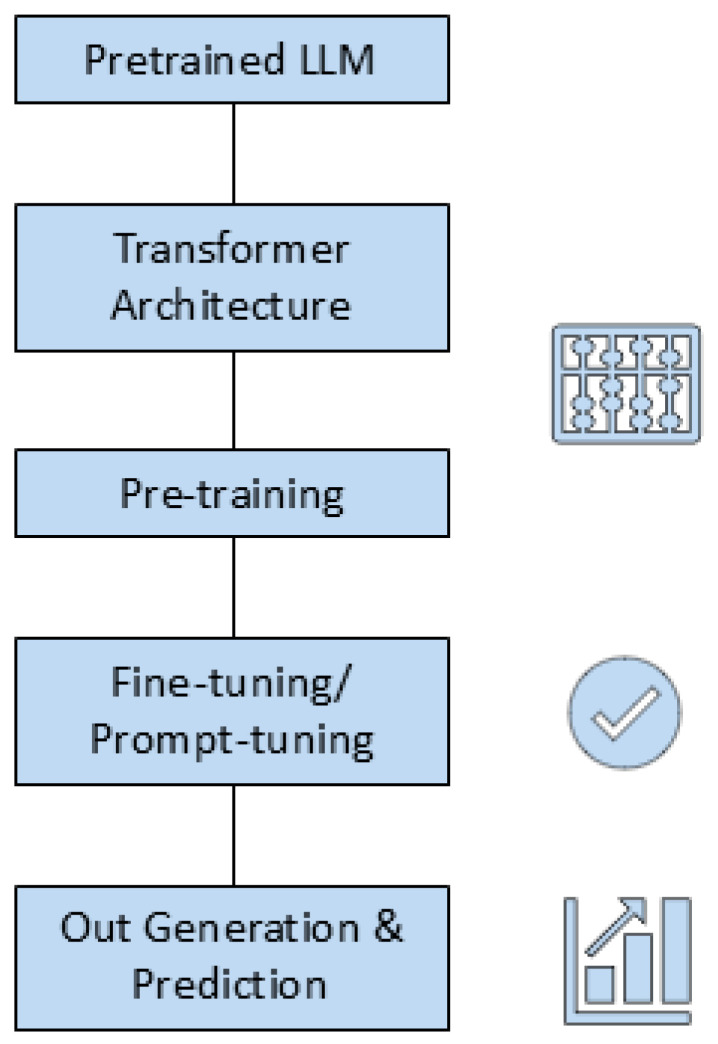
Large language workflow model for AMP prediction and design. Curated AMP corpora are tokenized and used to pretrain and fine-tune, with lightweight adapters and multi-task heads. For prediction, the model scored sequences with calibrated uncertainty, and generating promptable templates produced candidates, which passed through safety filters, i.e., hemolysis and toxicity, AF3, an active design to improve potency while minimizing risk iteratively.

Large language models (LLMs) can generate peptides by treating amino acid strings as “text’’ by training on protein sequences [194] (Table 6 and Table 7) (Figure 4). “AMP-GPT,” a trained GPT-2 model, has 12 transformer layers and 8 attention heads; the architectural framework was presented with a large peptide corpus [194]. Later, contrastive prompts, multi-stage fine-tuning, and reinforcement learning were employed to generate AMPs conditioned on target strains [194]. In an in vitro study, 17 of 18 generated peptides demonstrated bactericidal activity against Gram-negative bacteria., i.e., novel candidate (P076) had an MIC of 0.21 μM against resistant *A. baumannii* [194]. For in vivo validation, P076 was associated with lower bacterial loads within an animal lung infection [194].

Representation-led models with transformer frameworks, such as PeptideBERT [186,195], PHAT [196], SenseXAMP [197], and UniAMP [58], demonstrated discriminative performances by coupling task-specific heads, i.e., binary classification, MIC regression, hemolytic and toxicity properties, alongside protein-language-model embeddings. These architectural frameworks make them natural scoring platforms within closed-loop designs and facilitate test–learn workflows. Amongst these, attention-enhanced architectures, e.g., iAMP-Attenpred [198], added interpretable class-specific saliency and residual level attributions, connected sequential motifs to biophysical hypotheses, guiding safer and mechanism-aware edits.

AI methods, particularly deep learning, have been used to predict and generate sequences of antimicrobial peptides [199,200]. LLMs can be used to mine vast peptide sequence spaces and produce novel AMP sequences that can effectively target clinical and resistant superbugs [199,200]. Transformer architecture facilitated a better understanding of intricate relationships within protein sequences, thereby outperforming conventional techniques that struggled to deal with such complexities [199,200]. This demonstrates the transformative impact of AI, especially for drug design, highlighting the need for developing new antimicrobial agents.

A fine-tuned and pretrained model, such as ProtGPT2, predicts property-specific AMPs [201]. PeptideGPT, a fine-tuned version of ProtGPT2, generated proteins with desired physicochemical properties, demonstrating an AMP generation accuracy of up to 68 to 78%, highlighting a general approach to using large pretrained language models alongside bioinformatic filtering [201]. LLMs can encode complex sequence grammar and be fine-tuned for specific outcomes [194]. They usually require extensive training data and computational resources, as well as external screening to select novel candidates [194]. Therefore, LLM-based models can be promising for AMP discovery, especially when integrated with high-throughput validation, but they still depend on large language models [194].

Large language models alongside transformers act as primary scoring backbones [202], layered attention approaches can enhance variants for interpretability [21], and CNN hybrids retained descriptor-based platforms can facilitate calibration and diversity boosters [131], keeping exploration broader by drawing from great enumerative and cheminformatic efforts. Moreover, by reporting calibration, i.e., Expected Calibration Error (ECE) and Brier scores, alongside strains out of distribution (strains OOD), toxicity trade-offs, and prospective hit rates, these performance metrics can demonstrate robustness and explainable novel outputs for translation validation [203].

**Table 6:** Different currently available large language model-based discriminative models.

Models	Architectural Framework	Performance Metrics	Predicted Properties and Key Features
**a. Transformer-based LLMs**			
PeptideBERT	BERT (ProtBert), and MLP	AUROC 0.953 (property task, not AMP vs. non-AMP)	Pretrained on UniProt with fine-tuning to predict peptides. Predicted toxicity, stability and non-fouling [186,195].
PHAT	ProtTrans and MLP	Accuracy 93.2% (Q3 secondary structure)	Predicted efficacy based on physiological conditions. Predicted pH-dependent activity [196].
SenseXAMP	ESM-1b, transformer-based protein model	Accuracy 91.4% (AMP vs. non-AMP, balanced dataset)	It captured evolutionary conservation using embeddings with minimal feature engineering. Predicted antimicrobial activity [197].
TransImbAMP	BERT, and MLP	Balanced accuracy (96.85%), MCC (0.8)	It addressed dataset bias using sensitive learning approaches. Predicted antimicrobial activity [180,201].
UniAMP	UniRep, ProtT5, and deep neural network with transformer encoders	Accuracy (96.2%), AUROC (0.987)	Integrated protein-language insights within sequences to predict based on AMPs. Predicted antimicrobial activity [58].
**b. Attention-enhanced architectural DL models**			
iAMP-Attenpred	BERT (ProtBert) and MLP	Accuracy (94%) (binary classification)	It highlighted residual embeddings and managed length sequences. Predicted antimicrobial and cytotoxicity activity [198].
PepHarmony	ESM, GearNet (GNN), and MLP	High ACC/AUC/F1 across tasks; AUROC (0.972) (Peptide classification benchmark)	It integrated geometric graph features to predict 3D structures. Predicted antimicrobial, stability, and synergistic activity [204].
**c. DL hybrid models with engineered features**			
AMPFinder	ProtTrans, OntoProtein, and MLP	Accuracy (>95%) AMP Identification	It incorporated protein sequences for functional annotations. Predicted pathogen-specific antimicrobial properties [205].
FSLSME	ESM-1, MLP	Accuracy 92.7%	It used different libraries, i.e., hexapeptide, heptapeptide, and octapeptide libraries, for mining AMPs. Predicted antimicrobial activity [206].
**d. Deep learning hybrid models with CNN architecture**			
AMPDeep	BERT (ProtBert) and MLP	Accuracy 91.8% (hemolytic, toxicity)	Optimized bioavailability. Predicted safety profiles [184,207].
AI4AMP	Deep neural network (LSTM, CNN, Dense)	Accuracy (91.7%), AUC (>0.9). Precision (90%)	It had PC6 encoding methods to map sequences into a physicochemical vector. Predicted antimicrobial activity [131].
sAMP-VGG16	VGG-style convolutional neural network	Accuracy (94.3%)	It optimized adaptive layers by integrating LLB and using deep convolutional models for AMP classification [74,203].
**e. Other approaches**			
Ma & colleagues	BERT, ATT, LSTM, MLP	Accuracy 92.5%	It provided sequential AMP prediction. Predicted in vitro and in vivo efficacy and safety prediction [21].
Orsi & colleagues and Reymond	GPT-3, MLP	Accuracy 88–90% (toxicity/activity benchmark)	It provided sequential AMP prediction alongside stability and toxicity assay [208].
Zhang & colleagues	BERT, MLP	AUROC (0.965) Peptide classification across benchmark datasets	Using pLM embedding, it predicted the AMP spectrum. Predicted antimicrobial activity [209].

### 8.4. Multi-Model Hybrid Approaches for AMP Mining and Discovery

**Figure 5:**
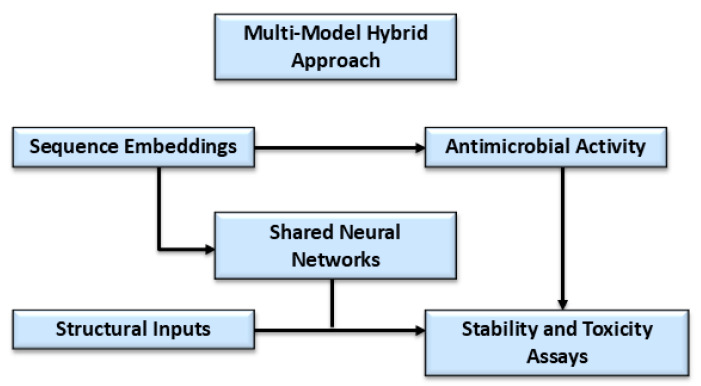
The block diagram shows a multi-model approach comprising processing sequences via LM embeddings plus physicochemical features, while structural branches process residue-contact graphs or 3D features. Representations are merged within shared fusion blocks and routed to multi-task heads for activity and MIC (regression), hemolysis, and toxicity (classification). Uncertainty estimates (ensembles dropout) and calibration layers support assay-prioritized decisions.

Beyond data mining and predictive modeling, recent studies have highlighted the importance of hybrid approaches combining deep learning and ensemble models, with experimental validations for AMP discovery [210] (Table 4 and Table 5) (Figure 5). Hybrid models demonstrate the trends of multimodal discriminators, i.e., leveraging large language models (LLMs) for sequence embeddings, structural graphs, attention, and ensemble architecture (Table 6 and Table 7) (Figure 5). A framework has been built showcasing how computational–experimental platforms can be utilized to identify novel AMPs from the human stomach’s microbiome, thereby reinforcing synergism between computational intelligence and lab-based experimentation regarding AMP discovery and validation [210].

#### 8.4.1. Hybrid Methods with Ensemble Frameworks

AMPredictor is a hybrid model, where deep Graph Convolutional Networks (GCNs) regressions bridge sequences, structural, and experimental validation for predicting AMP activity [46]. An architectural framework has been proposed, using GCNs and scoring peptide sequences, represented as graphs where nodes were amino acids (using ProtBERT, ESM-2 embeddings) and edges came from predicted contact maps, i.e., AlphaFold2, to predict the log-MIC values [46]. This hybrid model showed lower Root Mean Square Error (RMSE) (0.53) and higher Pearson correlation (0.71) for predicting MICs, indicating outperformance over CNNs, RNNs, and transformers [46]. In vitro and in vivo methods were used for 16 novel candidates regarding experimental validation, yielding stronger antimicrobial activity [46].

TransImbAMP, a BERT-like Transformer [202], a hybrid model possessed standard Transformer encoders, such as multiple attention layers and token embedding, producing 768-dimensional vectors per sequence, followed by multi-layer perceptron (MLP) output layers, thereby addressing two tasks, i.e., multi-label prediction for functional targets and binary AMP classification [202]. For larger curated datasets, the hybrid model demonstrated a balanced accuracy of 96.85% for binary AMP classification and 79.8% accuracy for functional targeting approaches, indicating the combination of a powerful Natural Language Processing (NLP) model with multi-label learning [202].

Deep neural networks can predict AMPs by learning their sequential features directly [135]. CNNs detect motifs by scanning peptide sequences and embedding their matrices [135]. An accuracy of up to 91% has been achieved for AMP classification using CNN and biLSTM, alongside Word2Vec amino acid embeddings [135]. Hybrid models such as ACEP combined CNN and LSTM and showed 92.5% accuracy based on sequence profile inputs [166], while AMPlify (Bi-LSTM with attention) achieved 93.7% [133].

#### 8.4.2. Hybrid Protein Language-Based Approaches

PGAT-ABPp leverages 3D structure, using AlphaFold2 via ColabFold folded sequences [211]. Two types of embeddings, i.e., spatial features from residue coordinates and a pretrained language-model embedding (ProtT5-XL-U50), capturing sequential contexts, were incorporated within the nodes [211]. This hybrid model, which fused language-model embeddings with predicted structural topology, outperformed 14 other methods in terms of accuracy, F1-score, and Matthews Correlation Coefficient (MCC), demonstrating improved performance [211].

Recent advancements have emphasized that bacteria-specific models will allow nuanced predictions against different pathogens. One study illustrated that developing the models to differentiate AMP activity against Gram-positive and Gram-negative (e.g., *A. baumannii*, *E. coli*) bacteria by integrating datasets specifically tailored for study groups aided in identifying novel peptides and understanding the variable mechanistic approaches that AMPs may employ depending on bacterial target type [212,213].

#### 8.4.3. Multi-Model Hybrid Approaches Based on Fusion Features

Multi-model hybrid approaches that fuse engineered descriptors alongside deep representations have accelerated the process of peptide discovery. AFP-MFL integrated multi-view sequential features to identify novel peptides with stronger accuracy, illustrating the importance of feature fusion when labels are limited [214,215]. Based on transformer embeddings, UniDL4BioPep [216,217] and Pang’s approach [202] coupled sequential language models with imbalanced training datasets to generalize outputs across diverse bioactive peptide classes. Later, UniproLcad combined multiple protein language models, such as UniRep, ESM-2, ProtBERT, and attention fusion, yielding novel AMP identification for cross-validation and independent testing [198].

To highlight feature-level fusion, certain models can integrate sequential physicochemical and structural features within one unified model [187]. AmpHGT fused multi-view graph representations, such as fragment, atom, and residue levels, alongside ESM-2 embeddings to predict pathogen-specific target and antimicrobial activity, allowing prioritization of peptides for wet-lab validation [187]. Ensemble architectural frameworks combining LLM embeddings alongside predicted secondary properties, such as charge and hydrophobicity, have been shown to optimize AMP physiochemical features, thereby guiding the synthesis of novel candidates with balanced efficacy and safety [218,219].

#### 8.4.4. Hybrid Large Language-Based Model

Large language models (LLMs) have been used to predict bactericidal and hemolytic activity, showing that tune-tuned GPT-3 models better predicted AMP properties as compared to specialized models [208]. GPT-based AI models, such as BroadAMP-GPT, integrated GPT-based generators with multi-stage screening and experiments to generate novel peptides (AMP-S13), followed by in silico filtering to detect broad-spectrum activity (i.e., 57% inhibition against “ESKAPE” pathogens) and higher stability and lower hemolysis, thereby promoting wound healing within a *Methicillin-Resistant S. aureus (MRSA)* animal model [220].

LLM-based GPT-3-AMP demonstrated that large language models predicted AMP and haemolytic activity directly from sequential texts, pointing to promptable, target-based pipelines, plugging within the closed-loop designs [194,208,220,221,222,223,224]. Transformer-classifier hybrid workflows range from pure sequence classifiers to closed-loop generative designs, blending domain-specific scoring and attention networks [208,220]. Future models must integrate rich features such as combining sequences and structural and physicochemical inputs, alongside design classification, via generative components [208,220].

At present, LLMs are embedded within different hybrid AI-driven pipelines rather than as isolated models. EBAMP has adopted a two-stage strategy where LLMs generated AMP candidates with broad-spectral activity, followed by physicochemical filtering [225]. Novel peptides with lower MICs and haemolytic activity have been reported using this technique. Likewise, ProteoGPT incorporated different specialized sub-LLMs with downstream property scoring systems, yielding effective AMPs against clinical and resistant pathogens [199,226,227].

**Table 7:** Different multi-models are used to identify and predict AMPs.

Model	Architecture	Predicted Properties	Key Features
**a. Multi-model hybrid methods have ensemble frameworks**			
AMP-EF Antimicrobial Peptide—Ensemble Framework	XGBoost, Bi-LSTM with attention	XUAMP (ACC 77.9%), CAMP (99.8%), XUAMP (AUC 0.894)	Multi-modal approaches provided strong generalization and higher performance. Predicted antimicrobial activity [228].
AMPpred-DLFF	ESM-2, CNN-based feature extractors	AUC (0.97)	Multi-model approaches synergized graph attention, protein-language embedding, and convolutional features for AMP predictions. Predicted antimicrobial activity [229].
AMPredictor	ESM-2, MLP, SVM	MIC regression (RMSE 0.535), PCC (0.71)	Using attention maps, it predicted key residues’ physicochemical descriptors [230,231].
PepMultiFinder	ML and multi-filter approaches	No global accuracy reported	Multi-model approaches to predict AMP efficacy and safety [232,233].
**b. Multi-model hybrid protein language-based approaches**			
FusPB-ESM2	ProBERT, ESM-2 embeddings, and Neural Network	Accuracy (0.983) (Independent test)	It fused two LLM embeddings for multi-functional representation and predicted multi-functional microbial activity [234,235].
PGAT-ABPp	ProtT5 embeddings and GAT	AUROC 0.983	Integrated geometric deep learning for 3D structural representation. Predicted efficacy and safety profile [211].
**c. Multi-model hybrid approaches based on fusion features and deep representation**			
AFP-MFL	Co-attention mechanism, MLP	ACC (96.8%), AUC (0.97)	ProtT5 and BLOSUM62 predicted physicochemical features, while co-attention and MLP explained multi-feature fusion and antimicrobial properties [214,215].
Pang’s Approach	Pre-trained BERT and MLP	ACC (96.9%), F1 (0.91) (AMP vs. non-AMP)	It predicted regression-based MICs and clinically relevant safety outputs [202].
UniDL4Biopep	ESM-2 embeddings and CNN	ACC (93.8%), MCC (0.875) bitter peptide datasets	Pretrained self-supervised model. CNN extracted spatial features from embeddings. Predicted antimicrobial and stability activity [216].
UniproLcad	UniRep, ESM-2, ProtBERT, 1D-CNN, Bi-LSTM with attention method	AUROC 0.982 (0.982 on XUAMP)	Multi-PLM fusion model with competitive accuracy and interpretability [198].
**d. Large language-based model**			
GPT-3-AMP	GPT-3 (generative) and SVM/RF (discriminative)	Activity: AUC (0.86), ACC (0.79) Hemolysis: AUC (0.89), ACC (0.84)	Uses GPT-3 to generate candidate peptides, using SVM/RF filters for antimicrobial activity [194,208,220,221,222,223,224].

## 9. Machine Learning Approaches Using Generative Frameworks

**Figure 6:**
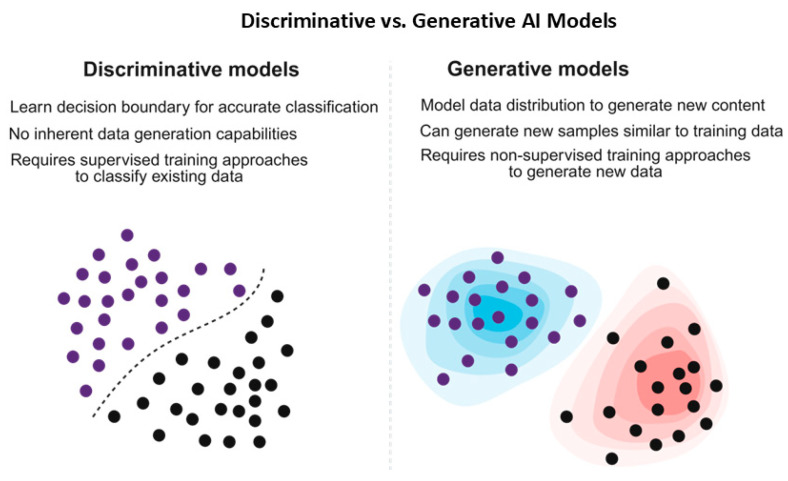
Key comparison between discriminative and generative models for AMP discovery and identification. Discriminative models are used to score existing sequences, emphasizing calibration, robustness, and triage for assays. On the contrary, generative models can be used to propose new sequences under constraints, i.e., higher stability and potency, with lower hemolysis and cytotoxicity. The image was created with BioRender (https://www.biorender.com, accessed on 12 November 2025).

Different deep generative models have been used to predict and design novel AMPs in silico (Figure 6). At present, studies have developed different machine learning approaches such as generative adversarial networks (GANs), variational autoencoders (VAEs), large language models (LLMs), and diffusion processes (Table 8).

### 9.1. Deep Learning Hybrid Models with GAN Architecture

Generative models have been used for the de novo design of AMPs [236]. One generative adversarial network (GAN) model that was trained on antibacterial peptide data for producing novel AMPs to combat antibiotic-resistant strains [236] identified a series of novel AMPs, which significantly expedited the discovery timeline with higher specificity for desired physicochemical properties [236]. Such approaches have not only accelerated the discovery and identification processes but also enhanced the potential for developing multi-purpose candidates capable of targeting various drug-resistant pathogens [237].

Current emerging trends, particularly in the realm of generative models, such as Multi-CGAN, aim to generate peptides not only to fulfil a single functionality but also to exhibit a range of properties mandatory for clinical outcomes [238]. Therefore, by training models on databases having specific physicochemical labels, their potential for creating multifaceted AMPs can be significantly increased, thus addressing the current gaps regarding traditional discovery methods with a focus primarily on single-attribute guidelines [238].

AMPGAN v2 is a bidirectional conditional GAN, comprising encoders, generators, and discriminators, allowing AMP editing and design alongside feedback loops [238]. For instance, Featurized Bidirectional Generative Adversarial Networks (FBGAN) can add an AMP classifier within the loops during the model training process; therefore, it can occasionally replace real data while generating sequences that can be labeled as active by classifiers, thereby guiding the generators [239]. The original Featurized Bidirectional Generative Adversarial Networks (FBGAN) classifier has been updated with a stronger version, using ESM-2 embeddings and k-mer features, which significantly improved performance, yielding generation quality as compared to previous models such as HydrAMP and AMPGAN [239].

GAN generators trained using Wasserstein-GP discriminators and Atchley factor-encoded peptides, alongside separate graph-convolutional regressors such as AMPredictor to predict MIC values [46], developed sequences which were scored by the regressors, and top candidates were filtered based on MIC < 10 μM, showing 24 novel peptides that demonstrated high bactericidal activity against multidrug-resistant strains [46]. Moreover, the top candidates, such as P001, P002, and P076, had MICs ranging from 0.20 to 0.47 μM against resistant *A. baumannii* [46]. Furthermore, P076 showed nonhemolytic properties within an animal model, highlighting that GAN models had 100% bactericidal hit rates amongst in vitro models [46].

Generative models have demonstrated new avenues for novel AMP design and discovery [239]. Deep generative approaches produced peptides having specific desired antimicrobial properties, thereby transforming the landscape of AMP discovery and prediction [239]. Therefore, investigations regarding AMP prediction must continue to refine currently available tools, leveraging AI to formulate effective therapeutic modalities against resistant pathogens.

Apart from prediction, the generation and testing of novel peptide candidates can benefit from machine learning (ML) methodologies. Deep learning can be utilized for the screening of bioactive peptides, speeding up the drug discovery process [240]. Certain deep learning generative models, such as LSTM_Pep, were used to produce de novo bioactive peptides, thereby leveraging existing AMP databases as training sets. The complementary screening frameworks, such as DeepPep, enabled rapid evaluation of generated peptides against specific therapeutic targets [240]. Moreover, iterative fine-tuning, generation, and screening approaches demonstrated a pragmatic AI pipeline to accelerate the discovery of novel antimicrobial peptides, which can be particularly beneficial given the ability to iteratively design novel peptides based on their predicted physicochemical and biological properties [240].

GANs have proven to be powerful models by learning rich sequence distributions and generating realistic and highly potent AMPs in combination with activity predictors [241]. However, they are harder to train and sensitive to several hyperparameters, often requiring careful discriminator design and auxiliary networks such as regressors and classifiers to enforce desired physicochemical properties [241]. In comparison to variational autoencoders (VAEs), generative adversarial networks (GANs) yield sharper and “peptide-like” sequences, thereby achieving higher success rates and diverse outcomes [241].

### 9.2. Variational Autoencoders (VAEs)

Variational autoencoder (VAE) models encode peptide sequences within continuous latent spaces and decode them to generate new sequences [242]. For conditional variational autoencoders (VAEs), the latent encoding is dependent on targeted physicochemical properties, thereby facilitating controlled generation [242].

Denoised conditional variational autoencoders (VAEs) use different physicochemical properties such as molecular weight, charge, isoelectric point, and hydrophobicity, embed them as conditional inputs during encoding and decoding processes, and improve the robustness of limited data using Kullback–Leibler (KL) divergence, a custom loss combining reconstruction and a transformer encoder–decoder, and a “property-preserving” alongside denoising inputs [242]. HydrAMP, a variational autoencoder (VAE)-based model, employed molecular dynamics-based filtering before synthesis, thereby optimizing diversity enhancement [218]. In an in vitro model, 9 of the 15 designed peptides exhibited high antibacterial activity, indicating these analogues were active prototypes that showed potent activity [218].

The application of more sophisticated and advanced computational frameworks, such as integration of generative artificial intelligence (AI) using variational autoencoders (VAE), allows for the design of novel peptides with desired antimicrobial properties by exploring a latent space of known sequences while filtering out ineffective sequences [237,243]. This approach allows for the optimization of peptide characteristics while mitigating the exhaustive and often resource-intensive trial-and-error process traditionally associated with peptide synthesis [243]. Therefore, exploration of latent spaces for peptide sequence generation has showcased the progressive trends towards comprehensively utilizing AI methods for peptide prediction and design [243].

Variational autoencoder (VAE) models incorporate multiple conditional variables and interpolate between sequences, therefore offering a stable framework with a well-defined latent space [242]. However, they may generate less distinctive outputs than generative adversarial networks (GANs), requiring careful design of the latent spaces [242]. For in silico methods, HydrAMP generated peptides with lower MICs. Variational autoencoders (VAEs) can generate novel AMP candidates with desired physicochemical properties, but have moderate hit rates without extensive filtering [243]. They might exceed within conditional designs but require screening steps such as MD filtering and sufficient training data to achieve higher success rates [243].

### 9.3. Diffusion Model

Diffusion, a score-based model, generates data by iteratively denoising random noise vectors within valid sequences [244]. AMP-Diffusion, a latent diffusion model, was built on the Evolutionary Scale Modeling (ESM)-2 protein language model and embedded peptides via ESM-2 within the continuous latent spaces and generated new latent vectors by applying diffusion processes by decoding them back to sequences [244]. This model generated sequences showing perplexity, diversity, and physicochemical properties identical to known AMPs [244].

ProT-Diff, a hybrid diffusion model coupled with pretrained ProtT5-XL-UniRef50 transformers as encoders and decoders between them, iteratively denoises random latent features into novel peptide encodings, which are later decoded by ProtT5 [245]. In an in silico method, 44 out of the proposed 45 candidates demonstrated better bactericidal activity against Gram-negative and positive pathogens [245]. In an animal peritonitis model, a novel candidate showed significantly reduced bacterial loads against resistant *E. coli* [245].

AMPGen, an autoregressive diffusion model, possessed axial attention over multiple sequence alignments (MSAs), alongside a Long Short-Term Memory (LSTM) scorer and XGBoost discriminators, incorporating evolutionary information, and took AMP-MSA as conditional inputs for generating target-specific peptides [246]. In an in vitro model, 38 out of the 40 synthesized peptides were antibacterial [246]. Amongst these, 31 novel AMPs were not within pre-existing databases and exhibited strong broad-spectrum activity [246].

Diffusion models can benefit from pre-trained language tools such as ESM-2 and ProtT5, which capture protein semantics. These models can identify novel regions within sequence spaces more efficiently than local search methods. However, they have higher computational costs, requiring specialized training and bigger sampling resources. Nonetheless, diffusion-based models such as AMPGen and ProT-Diff can yield potential novel AMPs with minimal filtering and demonstrate better in vivo efficacy compared to generative adversarial networks (GANs) [245,246].

### 9.4. Other Generative Models

Foundation-based models alongside various generative models broadened the design spacing beyond motif editing. Pep-based variational autoencoders (VAEs) have been used to demonstrate smooth latent manifolds for guided editing and MIC-based sampling, making toxicity avoidance tractable within lower datasets [247]. GAN frameworks delivered diverse proposals, from WGAN-GP systems for AMP generation to bifunctional AMP pipelines, coupled with generative adversarial networks (GANs) and activity regressors, showing in vitro and in vivo validation [248].

To highlight translation implications, an activity predictor was integrated before generative models to incline toward non-hemolytic designs, highlighting the importance of multi-objective control [249]. Latent diffusion models, i.e., AMP diffusion, were tailored to proposed novel potent peptides with low toxicity in vivo, while AMPGen fused evolutionary methods before diffusion to target disordered, AMP-like sequences, eluding fold-centric designs [250].

**Table 8:** Different generative methods for AMP discovery and prediction.

Generative Methods for AMP Discovery			
Models	Architectural Frameworks	Control Generation	Key Features and Performance Metrics
**a. GAN architectural frameworks**			
AMP-GAN	GAN (Generator and Discriminator)	Random latent vectors without explicit conditioning	Antimicrobial and cytotoxicity assays [251,252].
AMPGAN v2	BiCGAN (Bidirectional Conditional GAN)	Binary vectors for targeting microbes and mechanisms	Antimicrobial assays. Metrics: Validity (95%), Novelty (94%), Uniqueness (100%) [251].
dsAMP and dsAMPGAN	CNN Attention, BiLSTM, transfer learning models		AMP’s prediction. Metrics: Accuracy (95%), F1 (0.94) [59].
FBGAN	GAN and ESM-2	Controlled and conditioned generation	Antimicrobial, hemolytic and cytotoxicity assays. Metrics: AUROC (0.92) [104,253].
WGAN-GP	WGAN-GP	AI4AMP and classifiers for in silico	Predicted novel peptides using methods like PC6, based on physicochemical properties [236].
Multi-CGAN	cGAN	Conditional generation	Antimicrobial and cytotoxicity assays [254].
**b. VAE and latent spaces-based frameworks**			
CLaSS (Controlled Latent Attribute Space Sampling)	WAE	Discriminator-guiding filtering	In vivo models using Antimicrobial, hemolytic and cytotoxicity assays. Metrics: Precision (90%) for desired sampling [255].
LSSAMP	Vector quantized VAE	Latent space sampling	Predict Antimicrobial, hemolytic and cytotoxicity assays. Metrics: Accuracy (91.7%) [255,256].
PepVAE	VAE	Latent space sampling	Microbial activity. Metrics: Validity (>95%), Novelty (80%) [247].
**c. Diffusion-based framework**			
AMP-Diffusion	Structurally guided diffusion model	Positive learning, using discriminator-guiding filtering	Microbial and cytotoxicity assays (in vivo). Sequence validity (97%) [237,257].
Diff-AMPs	Diffusion	Discriminator-guiding filtering	AMP’s prediction. Metrics: AUROC (0.94) [258].
ProT-Dif	Protein language diffusion	Condition generation with discriminator-guided filtering. Positive-only learning	De novo generation of novel AMP sequences. Metrics: Validity (98.3%), Novelty (99%) [245].
MMCD	Diffusion model based on discriminators	Conditional generation, contrastive learning	Predict microbial, hemolytic and cytotoxicity assays [259,260].
**d. Multi-objective evolutionary or genetic optimization models**			
AMPEMO	Genetic algorithm	Discriminator-guiding filtering	Antimicrobial activity [261,262].
MODAN	GAN and RL	Bayesian optimization	Antimicrobial, hemolytic assays. Multi-objective score improvement >30% over baseline [263].
MOQA Multi-CGAN QMO (Multi-Objective Quantum Annealing)	Binary VAE, Multi-generator CGAN	D-Wave quantum annealer, with conditional generation	In vivo models to predict Antimicrobial, hemolytic and cytotoxicity assays [264].
M3-CAD	cVAE	Conditional generation using discriminator-guided filtering	In vivo models to predict Antimicrobial, hemolytic and cytotoxicity assays [102].
QMO	WAE	Zero-order optimization with gradient descent	Optimized materials, i.e., drug-likeness and solubility [265].
**e. Transformers or an RNN-based hybrid framework**			
AMPGen	Autoregressive diffusion model, XGBoost discriminator, and STM	MSA-conditional generation	Microbial activity. Metrics: Validity (94%), Novelty (96%) [246,250].
AMPTrans-LSTM	LSTM, and transformers	Learning using protein databases	Antimicrobial activity [158].
HydrAMP	cVAE-GAN hybrid	Conditional generation	Antimicrobial, hemolytic assays. Metrics: AUROC (0.93) [243].
**f. Other generative models**			
Buehler & colleagues	GNN	Conditional generation	Physicochemical properties prediction [266].
Cao & colleagues	GAN	Discriminator-guiding filtering	Antimicrobial activity [248].
Capecchi & colleagues	RNN	Positive learning, using discriminator-guided filtering	Antimicrobial and hemolysis assays [176].
Dean & colleagues	VAE	Latent space sampling	Antimicrobial activity [247].
Ghorbani & colleagues	VAE		AMP prediction. Metrics: AUROC 0.90 [267].
Jain & colleagues	GFlowNets and active learning	Active learning	AMP prediction [268].
Pandi & colleagues	VAE	Discriminator-guiding filtering	Antimicrobial, hemolytic and cytotoxicity assays. Metrics: Validity 96% [269].
Renaud & colleagues	VAE	Latent space sampling	Physicochemical properties prediction [249].
Zeng & colleagues	PLM and BERT	Discriminator-guiding filtering	Antimicrobial activity. Metrics: Accuracy 92% [270].

## 10. Evolutionary and Genetic Algorithms for AMPs Prediction

Genetic and evolutionary algorithms have offered alternative approaches to explore AMPs along with multiple objectives such as antimicrobial activity, stability, and toxicity. Incorporation of evolutionary algorithms along with AI-based approaches has accelerated AMP discovery [271]. These techniques have helped researchers in investigating and optimizing the peptide sequence spaces through a closed feedback loop, combining computational predictions and in vitro assays to iteratively improve peptide candidates [271]. This synergy has expedited the identification of novel candidates and enhanced the basic understanding of the peptides’ design principles that regulate their activity [271].

A multitude of studies have reported successful peptide design approaches by incorporating diverse machine learning models, alongside genetic algorithms [272,273]. This hybrid approach led to the identification and characterization of novel peptides with proven bactericidal activity against *Staph. epidermidis*, illustrating the robustness of AI strategies for peptide discovery [272]. Similarly, Moretta et al. employed machine learning algorithms for analyzing antimicrobial peptides, which were identified from the Black Soldier Fly, highlighting their potential insights to discover natural antibacterial compounds [273].

Non-dominated sorting genetic algorithm-II (NSGA-II) can predict AMPs against *Staph. aureus*, thereby optimizing physicochemical properties, i.e., hydrophobicity, stability to enhance stability, efficacy, and safety [274]. Non-dominated sorting genetic algorithm-II (NSGA-II) was coupled with two neural network models to score candidates’ antimicrobial effectiveness [274]. Later, evolutionary algorithms efficiently identified and predicted Pareto-optimal peptides having improved stability, thereby accelerating ‘de novo’ AMP designs without compromising activity [274]. Therefore, multi-objective frameworks can be incorporated to explicitly address immunogenicity outcomes [274].

Genetic algorithms can be combined with interpretable ML techniques to customize AMPs [272]. ‘DNA codons’ were illustrated as peptides to investigate sequence variations, with defined rules to explain antibacterial fitness boundaries, by incorporating in vitro bacterial assays into the loop; novel candidates generated by the genetic algorithms (GAs) were synthesized and evaluated against *Staph. epidermidis* [272]. The closed-loop “directed evolution” approach identified novel candidates with limited aggregation propensity [272].

Evolutionary methods can be integrated with high-throughput in silico approaches to propose sequences, either to estimate MIC using regressor models or to predict selectivity indices [275,276]. Hemolytic models predict toxicity to discard potentially harmful candidates before experimental validation [275,276]. Novel candidates can be synthesized and validated through in vitro and in vivo methods using multi-model models such as BroadAMP-GP [220]. Therefore, integration of machine learning alongside experimental feedback approaches can predict novel AMPs with desirable physicochemical features, highlighting Evolutionary Algorithm (EA)-driven designs from genetic and evolutionary algorithms, which could be used as a key strategy for optimizing novel AMPs [275,276].

## 11. Perspective on Evolution of ML Approaches for AMP Discovery and Optimization

Evolution from classical machine learning to deep learning approaches and large language models has revolutionized the computational toolkit for AMP discovery [277,278]. However, increasing architectural frameworks does not guarantee better performance metrics [277,278]. At present, there is no single machine learning architectural framework that is optimal for all AMP mining, identification, and characterization. It is highly dependent on the types of tasks and currently available data resources [277,278].

The authors’ perspectives are that task-matched model selection will be more beneficial than automatic progression based on complex architectural frameworks. Classical models, RF, SVM, and XGBoost, are acceptable for smaller, imbalanced datasets for rapid AMP classification, while deep learning models, such as CNNs, LSTMs, and transformers, outperform these on larger sequential labeled datasets that are available for learning long-range residual dependencies [36,39,273]. Structural tasks usually require 3D-aware geometric deep learning approaches for receptor-specific designing and membrane-interaction modeling [277]. Decisions regarding the selection of different architectural frameworks vary with available datasets and modality and predictive outcomes [194].

Certain models, such as EBAMP and AMPGen, when used, demonstrated that architectural framework selection was task-driven, i.e., RL-based and diffusion models were preferred for de novo AMP generation, whereas transformer-based MIC regressors succeeded for efficacy predictions, and ensemble models were favored for safety profiles alongside multi-objective screening when interpretability and stability were critical [116,161,225]. Earlier genetic and evolutionary approaches demonstrated better outcomes regarding multi-objective AMP optimization, but their performance metrics were hampered by limited available datasets and noisy biological data [275,276].

To support this principle, two consolidated tables (Table 9 and Table 10) summarize different machine learning models with reported wet-lab, in vitro, or in vivo validations. These highlight their predicted outcomes for experimentally validated novel peptides, which is an essential consideration for translational impacts, prioritizing models that can offer real-world performance for antimicrobial performance, not just in silico validity.

AMP workflows now span diverse objectives, including structure-based design and classification, target specificity, MIC regression, and toxicity predictions [7,199,227]. Decision frameworks will help non-experts select the correct model classes depending on research queries and computational resources [248,262]. Model evolution has expanded the toolkit for AMP discovery, but progress can be made when architectural choices are aligned with biological context and data quality, and not complexity alone [248,262]. Therefore, the decision tree will substantially help microbiologists and computational practitioners regarding mapping applications for recommending architectures, aligning with the best practical implications for AMP discovery and optimization [248,262].

**Table 9:** Different machine and deep learning models for AMP predictions with experiment-based evidence.

Models	Architectural Frameworks	Experimental Evidence
AmPEP	RF (classical ML)	Validated on curated AMP datasets [114].
AMPpred-EL	Ensemble ML	Improved AMP identification across multiple datasets [110].
AMP-BERT/LMPred	Transformer	BERT embeddings improved AMP prediction accuracy [152,153].
AMPlify	Attentive DL	Peptides against WHO-priority pathogens [132,133].
BERT-AmPEP60	Transformer MIC regressor	Predicted MICs were experimentally confirmed [111].
Deep-AmPEP30	DL (CNN)	Prioritized short peptides for testing [138].
De-extinction/APEX	DL and evolutionary	Extinct AMPs validated in vitro/in vivo [24,26].
DMAMP	Multi-task DL	Peptides with multi-functional activities [141].
MBC-attention	Multi-branch CNN	MICs correlated with in vitro outcomes [148,149].
Macrel-type pipelines	DL and rule-based	Predicted AMPs experimentally validated from human microbiomes [170,171].
Non-hemolytic design	ML/DL optimization	Generated peptides with lower hemolysis [176,189].
PGAT-ABPp/sAMP-GAT	PLM and GNN	Peptides with improved accuracy [29,150].

**Table 10:** Generative and large language model-based frameworks for AMPs predictions with experimental validation.

Model	Architectural Frameworks	Experimental Evidence
AMPGAN v2	GAN	GAN-generated peptides against pathogens [251].
AMP-Diffusion	Diffusion and PLMs	Generated AMPs were validated [244,258].
AMPGen	Evolutionary and diffusion	Peptides against Gram-negative bacteria [250].
Diff-AMP/ProT-Diff	Diffusion frameworks	Peptides with micromolar MICs [245].
FBGAN	Generative and feedback	Feedback loop yielded peptides with better efficacy [253].
GA and ML approaches	Genetic and ML	GA-designed peptides with improved activity.
GPT-3-AMP/ Peptide-GPT	Foundation LLM and fine-tuning	Generated peptides showed good experimental activity.
Latent diffusion LMs	LLM and diffusion	Generated peptides with confirmed activity [237].
LLM-AMP frameworks (EBAMP, BroadAMP-GPT)	LLM-based design	LLM-generated peptides validated against clinical strains [220,225].
Multi-CGAN	Conditional GAN	Generated peptides with good efficacy and safety [254].
PepVAE	VAE	VAE-generated AMPs were active in vitro [247].

## 12. Challenges and Future Directions

**Figure 7:**
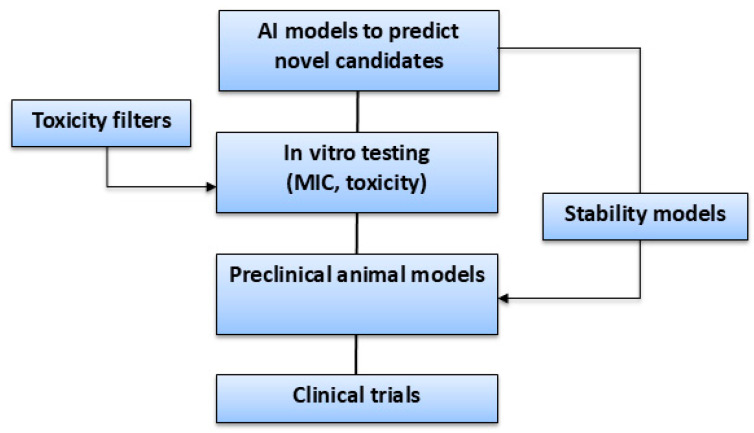
Artificial intelligence (AI) models showing a translational flowchart for mining and predicting novel candidates, which comprises advanced artificial intelligence approaches, along with different in vitro laboratory methods, i.e., MIC and toxicity assays, followed by preclinical (in vivo) established animal models, and eventually validated by human clinical trials, incorporating feedback loops in between, such as toxicity and stability filters.

There were several challenges associated with AMP discovery. A primary concern is the accuracy of these models’ predictions, which depends on high-quality training data. In this regard, databases such as the Database of Antimicrobial Activity and Structure of Peptides (DBAASP) play a crucial role by providing the datasets essential for ML model training and validation [279]. Furthermore, pharmacokinetics issues such as peptide stability, susceptibility to both enzymatic and non-enzymatic degradation, and the balance between bactericidal activity and cytotoxicity remain essential for the clinical implications of AI-predicted peptides [138] (Figure 7).

Certain challenges remain when standardizing artificial intelligence approaches across different studies. Various predictive models have proven that some machine learning methods may excel at predicting AMPs, but there is inconsistency within the methodologies, highlighting their difficulties in benchmarking their performances against each other [280]. Therefore, standardized datasets and enhanced interoperability can improve the overall reliability of these predictive models.

Predictive models that utilize features derived from the structure and function of peptides, such as Recurrent Neural Networks (RNNs) alongside AlphaFold models, can be trained on databases possessing extensive peptide sequences along with associated antimicrobial properties, thereby facilitating the identification and prediction of novel non-hemolytic peptides [179,279]. These approaches have streamlined the discovery of peptides having desirable biological properties, whilst minimizing the adverse effects associated with conventional antimicrobial therapies [179,279].

Future directions should focus on AI-driven AMP discovery and identification, which can benefit from multi-faceted approaches that incorporate insights from bioinformatics, structural biology, and proteomics (Figure 7). By integrating different fields, this could pave the way for developing peptides with complex functionalities suitable for clinical implications. Furthermore, the optimization of various physicochemical properties, such as stability, effective bactericidal activity, and non-hemolytic potential using advanced artificial intelligence technology will facilitate exciting avenues for ongoing research related to novel AMP identification and prediction [178].

## 13. Conclusions

Artificial intelligence is fundamentally revolutionizing the landscape for antimicrobial peptide discovery. Therefore, by harnessing different machine learning approaches along with advanced computational techniques, researchers can uncover several novel peptides that hold promise for tackling the global challenges associated with antibiotic-resistance trends. As these technological approaches evolve, they will lead to significant advancements regarding drug discovery, design, and development, thereby shaping the future of conventional antimicrobial therapies to combat drug-resistant pathogens.

In conclusion, computational intelligence can play a decisive role in novel antimicrobial peptides discovery by streamlining the identification, prediction, and design of potential AMPs. Therefore, the integration of sophisticated machine learning models, coupled with extensive peptide databases and in vitro and in vivo experimental validations, can make it possible to explore and identify a rich landscape of antimicrobial peptides, heralding a promising alternative novel therapeutic strategy for combating resistance, particularly against Gram-negative pathogens.

## Figures and Tables

**Table 1:** Comparison between different machine learning approaches.

Features	Supervised	Unsupervised	Reinforcement
Definition	Learns from labeled data resources.	Learn and identifies patterns from unlabeled data.	Learn via interaction with the environment.
Type of data	Requires labeled data.	Requires unlabeled data.	Learning from the environment, there is no predefined data.
Barriers	Barriers, such as classification, regression.	Clustering and association are the barriers.	Performs sequential decision-making.
Supervision approach	Requires external supervision.	Does not require external supervision.	Learn from feedback responses.
Algorithm	Including Random Forest (RF), K-Nearest Neighbours (kNNs), Support Vector Machines (SVMs), neural networks, and decision trees.	Includes K-Means, Principal Component Analysis (PCA), autoencoders.	Includes Q-learning, Deep Q-Network (DQN), and State-Action-Reward-State-Action (SARSA).
Outcomes	Predicts outcomes with accuracy.	Discovers hidden patterns.	Optimize actions for maximum rewards.
Limitations	Requires larger and well-labeled datasets. Performance degrades when applied to other peptides not represented in the training data	Misleading classification if data is not labeled. Clustering or embeddings may group peptides based on artefactual similarity rather than biological function.	Highly depends on well-designed rewards. Poorly shaped rewards can generate biologically implausible peptides.

**Table 2:** Comparison between different machine learning approaches based on task categorization.

Approach	Primary Task	Strengths	Limitations
Conventional ML	Functional grouping is based on non-AMP vs. AMP classification.	Easy to interpret with low data requirements, fast to train.	Not ideal for novel peptides due to limited sequential modeling.
Deep Learning	Predicts activity based on motif learning.	Shows nonlinear sequential activity relationships.	Low transparency and larger datasets.
Recurrent Models	MIC regression, based on order-dependent prediction.	MIC predictions.	Training instability with longer sequences.
Regression Models	Potency ranking based on MIC value predictions.	Favors dose estimation and prioritization.	Outcomes depend on dataset quality.
Transformer-based Models	Multi-task predictions (target specificity, activity, toxicity).	State-of-the-art accuracy.	Larger computational resources.
Ensemble/Hybrid Models	Robust AMP classification, alongside multi-feature fusion.	Integrates physicochemical features and higher stability.	Harder to interpret due to complexity.
Large Language Models	Species-specific peptide generation and MIC predictions.	Infers structural and functional constraints.	Requires higher computing.
Protein Language Model Hybrids	Multi-target AMP profiling.	Highly transferable and learns embeddings biologically.	Needs pathogen-specific tunings.
Generative Models	De novo AMP designs.	Optimize novel AMPs beyond natural diversity.	Needs robust functional scoring.
Evolutionary and Genetic Algorithms	Iterative optimization of selectivity, potency, stability	Multi-objective AMP engineering	It depends on the fitness predictor’s accuracy

**Table 3:** Comparison between different data modalities.

Modality	Descriptors	Machine Learning Approaches	Applications
0D (Global Features)	Fixed-length descriptors not depending on sequence order	Classical ML, ensemble models	Global assembly with AMP vs. non-AMP screening, toxicity prediction.
1D (Sequential Data)	Linear amino acid sequences are encoded as residues.	CNNs, LSTM, BiLSTMs, transformers.	Generative sequential designing, with MIC and activity prediction, target-pathogen profiling.
2D (Matrix-Like Representations)	Pairwise residue–residue matrices, contact maps.	CNNs, hybrid CNN-transformers.	AMP vs. non-AMP classification by capturing spatial-like physicochemical patterns.
3D (Spatial Information)	Three-dimensional atomic coordinates.	GNNs, 3D CNNs, docking-integrated ML.	Structure-based AMPs design predicting receptor-specific and membrane-interaction features.

## Data Availability

No new data were created or analyzed in this study.

## Abbreviations

The following abbreviations are used in this manuscript:

*A. baumannii*	*Acinetobacter baumannii*
ACEP	Antimicrobial Peptide Recognition
ADAM	A Database of Anti-Microbial Peptides
AI	Artificial Intelligence
AMPs	Antimicrobial Peptides
AMR	Antimicrobial Resistance
AMPDB V1	Anti-Microbial Peptide Database Version 1
ANNs	Artificial Neural Networks
APD-3	Antimicrobial Peptide Database-3
ATT	Attention Mechanism
AUROC	Area Under the Curve
B-AMP	Biofilm-AMP
BERT	Bidirectional Encoder Representations from Transformers
Bi-LSTM	Bi-directional Long Short-Term Memory
CAMP	Collection of Anti-Microbial Peptides
CNN	Convolutional Neural Network
DA	Discriminant Analysis
dbAMP	Database of Antimicrobial Peptides
DBAASP	Database of Antimicrobial/Cytotoxic Activity and Structure of Peptides
DF	Deep Forest
DL	Deep Learning
DPL	Database of Peptide Ligands
DRAMP	Data Repository of Antimicrobial Peptides
*E. coli*	*Escherichia coli*
ECE	Expected Calibration Error
ESM	Evolutionary Scale Modeling
FBGAN	Featurized Bidirectional Generative Adversarial Networks
GANs	Generative Adversarial Networks
GCN	Graph Convolutional Networks
GNN	Graph Neural Network
GRU	Gated Recurrent Unit
GRAMPA	Giant Repository of AMP Activities
GPT-3	Generative Pre-trained Transformer-3
IAMPE	Integrated Antimicrobial Peptide Estimator
InverPep	Invertebrate Peptides Database
KL	Kullback–Leibler
kNNs	k-Nearest Neighbours
*K. pneumoniae*	*Klebsiella pneumoniae*
LAMP2	Linking Antimicrobial Peptides-2
LGBM	Light Gradient-Boosting Machine
LLMs	Large Language Models
LSTM	Long Short-Term Memory
MCC	Matthews Correlation Coefficient
MIC	Minimum Inhibitory Concentration
MLP	Multi-Layer Perception
ML	Machine Learning
modlAMP	Molecular Design Laboratory’s Antimicrobial Peptides
MRSA	Methicillin-Resistant *Staph. aureus*
MSA	Multiple Sequence Alignments
NLP	Natural Language Processing
NSGA-II	Non-Dominated Sorting Genetic Algorithm-II
*P. aeruginosa*	*Pseudomonas aeruginosa*
PGAT-ABPp	Position-aware Graph Attention for Antibacterial Peptides
PSEAAC	Pseudo Amino Acid Composition
QSVM	Quantum Support Vector Machine
RF	Random Forest
RMSE	Root Mean Square Error
RNN	Recurrent Neural Network
*S. aureus*	*Staph. aureus*
SAR	Structure–Activity Relationships
SVM	Support Vector Machine
StAPD	Stability-Aware Peptide Database
VAEs	Variational Autoencoders
YADAMP	Yet Another Database of Antimicrobial Peptides
